# Exploring chemical diversity in *Glycine max* cultivars: A multivariate approach in the search for bioactive compounds against *Spodoptera cosmioides*

**DOI:** 10.3389/fpls.2022.987782

**Published:** 2022-09-02

**Authors:** Maria Clara Santana Aguiar, Marcelo Mueller de Freitas, Carlos Alessandro de Freitas, Arlindo Leal Boiça Júnior, Renato Lajarim Carneiro, Maria Fátima das Graças Fernandes da Silva, João Batista Fernandes, Moacir Rossi Forim

**Affiliations:** ^1^Laboratory of Natural Products, Department of Chemistry, Universidade Federal de São Carlos, São Carlos, Brazil; ^2^Laboratory of Plant Resistance to Insects, Department of Agricultural Sciences, Universidade do Estado de São Paulo, Jaboticabal, Brazil; ^3^Laboratory of Applied Chemometrics, Department of Chemistry, Universidade Federal de São Carlos, São Carlos, Brazil

**Keywords:** *Glycine max* L., different genotypes, metabolomic analyses, resistance biomarkers, *Spodoptera cosmioides*

## Abstract

Soybean crop is regulated by abiotic and biotic stresses with great potential in reducing grain yield and quality. The selection of resistant cultivars is a promising approach for mitigating these damages. We evaluated the chemical profile of *Glycine max* leaves from different cultivars in order to explore their defense mechanisms against *Spodoptera cosmioides* caterpillars. We optimized solid–liquid extraction techniques using ultrasound bath and static headspace extraction. Additionally, we developed an approach based on liquid and gas chromatography for analyzing the chemical profile of *G. max* cultivars. The principal component analysis allowed the classification of transgenic cultivars, which are classified as susceptible to *S. cosmioides*, from those obtained by genetic improvement and resistant to the insect. Differences were observed in the abundance of phenolic glycosides, lipids, aldehydes, and alcohols. More specifically, *S. cosmioides* resistant cultivars presented molecules related to the jasmonic and salicylic acid pathways. Such data can contribute to a molecular understanding of phenotypic diversity in soybean cultivars, from plant quality to resistance mechanisms and adaptation, to environmental stress and herbivory.

## Introduction

Plants continually develop chemical adaptations in order to improve their survival and reproduction, such as reducing the impact of predatory herbivores and phytopathogens, or attracting pollinators (Arimura et al., 2005). As such, they defend themselves and communicate through secondary metabolites in order to compensate immobility (Van Arnam et al., 2018). Under environmental stimuli, plants can produce more than 200,000 different metabolites. This diversity in chemical nature, physics, and concentration of magnitudes offers a challenge for chemical ecology studies in plant resistance mechanisms (Dunn and Ellis, 2005). A comprehensive analysis of such low molecular weight compounds (<1.5 kDa) from a given botanical system can be a complex endeavor, since the metabolomic profile is directly or indirectly involved in all aspects of cellular functions (Bi et al., 2013; Kumar et al., 2017; Sharma et al., 2018).

The characterization of chemical diversity involves a variety of stages. Sample preparation is the first stage of the research (Nováková and Vlčková, 2009), and their quality is fundamental in acquiring the results (Huie, 2002). Therefore, an optimized and standardized protocol for efficient metabolite extraction is needed in order to ensure reproducibility, especially when performing experiments that collect biologically significant information to help us understand a phenotype (Bi et al., 2013).

The technique used to detect molecules is also an essential stage in determining the compounds related to plant chemical adaptations (Dunn and Ellis, 2005; Cox et al., 2014). Among detection techniques, we can highlight Mass Spectrometry (MS). With MS, it is possible to detect and identify a variety of molecules for sensitive and selective analyses (Dunn and Ellis, 2005; Kumar et al., 2017). As such, combination of MS with Gas and Liquid Chromatography is one of the main analytical platforms for comprehensive plant metabolome profiling (Dunn and Ellis, 2005).

The study of flavonoid biosynthesis in soybean leaves is a successful application of chromatography separation and MS identification. Gomez et al. (2018), Li et al. (2017), and Song et al. (2014) demonstrated that flavonoid biosynthesis, isoflavones mainly, is not only related to plant development stages. It also interferes with plant resistance to herbivory and saline stress. Gomez et al. (2018), Hyeon et al. (2020), and Zhang et al. (2016) also evaluated the influence of soybean varieties such as cultivated, wild, and those obtained by genetic improvement on the phenylpropanoid pathway. Their results showed that cultivated crops induced alternative metabolic pathways compared to wild varieties in order to defend themselves against environmental stress and herbivory.

Considering this context, we have been using soybean cultivars (*Glycine max* L.) as a plant matrix model. Soybean is one of the main crops in production volume and is cultivated across the globe due to its high nutritional value in human food and animal feed as well as its application as a biofuel (Hartman et al., 2011). Nevertheless, soybean cultivation has been infested by caterpillars of the genus *Spodoptera*, which damages its leaves and pods (Bueno et al., 2011; Hartman et al., 2011; Bernardi et al., 2014). This interferes with pod formation and growing of the grains. The photosynthetic process is also limited when defoliation occurs, which interferes in growth by causing atrophy and even plant death. These processes have encouraged the application of synthetic insecticides (Bueno et al., 2011; Hartman et al., 2011; Bernardi et al., 2014). This has more recently led pest management programs to study eco-friendly strategies for *Spodoptera* sp. control (Bueno et al., 2011; Hartman et al., 2011) through understanding how molecules produced by plants may affect insect pest biological parameters. These molecules can be used as options for managing insects (Kumar et al., 2017; Sharma et al., 2018). However, there is no reported data on the chemical profiling of volatile and nonvolatile compounds, nor a comprehensive characterization of biological answers from different *G. max* cultivars under environmental stress and herbivory.

Our objective was to evaluate the differences in the chemical profiling of different *G. max* cultivar leaves and correlate the chemical data to resistance against *Spodoptera cosmioides* caterpillars. At the same time, we also evaluated the protocols highlighting optimal conditions for solid–liquid extraction, solid–liquid extraction assisted by ultrasound bath, and headspace extraction in order to characterize nonvolatile and volatile metabolites present in *G. max* leaves. Multivariate analyses, such as principal component analysis (PCA) and hierarchical cluster analysis (HCA), were also used in order to provide insights into the constitutive resistance of *G. max* cultivars and highlight resistance biomarkers to *S. cosmioides*.

## Materials and methods

### Reagents

Stock standard caffeine solution was prepared in water at a concentration of 100 μg⋅ml^−1^ and stored at 8°C. The same procedure was used preparing a menthol solution 500 μg⋅ml^−1^ in glycerol (Arora Produtos Químicos, São Marcos, Brazil). Caffeine and menthol were used as internal standards for Liquid and Gas Chromatography, respectively. Methanol LC–MS (Honeywell, North Carolina, United States), glycerol (Synth, São Paulo, Brazil), and ultrapure water (Milli-Q, Millipore, Merck KGaA, Darmstadt, Germany) were used as solvents. Formic acid (LC–MS, Fluka, Missouri, United States) was also used in the liquid chromatography mobile phase.

### Plant and insect materials

*Glycine max* plants in the reproductive stage (10 weeks) were used in this study. Ten different cultivars were evaluated: PI 227682, P98Y11 RR, UFUS Xavante, UFUS Milionária, UFUS Impacta, UFUS Carajás, UFUS Capim Branco, CD 208, Anta 82 RR, and M 8230 RR. Plants were grown in polyethylene vases of 3.0 L containing a 3:1:1 soil substrate of dystrophic red latosol, sand, and organic compound, respectively. The soil was derived from alkaline rocks, with an average density of 77 g⋅cm^3^, pH 5.5, containing calcium, magnesium, potassium, and 2.4% (w/w) organic matter. Ten seeds were planted in each pot. Having more than three germinated seeds, we discard the surplus seedlings. Plants were kept in a greenhouse sealed with anti-aphid mesh and watered daily with tap water as needed in uniform quantity among plants. For the experiments, plants were subjected to field conditions under a natural photoperiod and an average temperature of 32°C during the growing season. Cultural practices such as fertilizer application were not used in order to avoid the detection of false resistances.

*Glycine max* plants were harvested in the experimental laboratory located at the Department of Agricultural Sciences, São Paulo State University, in the municipality of Jaboticabal, São Paulo State, Brazil (21°14′25″S and 48°17′21″W) under the supervision of Professor Arlindo Leal Boiça Júnior. We selected *S. cosmioides* caterpillars (third instar) maintained in a laboratory in the same department, and fed an adapted artificial diet composed of a pinto bean (*Phaseolus vulgaris* Pinto Group; 7.35% w/v), wheat germ (5.90% w/v), soybean protein (2.90% w/v), and casein (2.20% w/v) mixture in water. Ascorbic acid (0.35% w/v), sorbic acid (0.17% w/v), formaldehyde (0.14% w/v), and tetracycline (0.007% w/v) were also added. Caterpillars were maintained under laboratory conditions at 25 ± 2°C, 70 ± 10% relative humidity, and under a photoperiod of 12 h.

### Collection of *Glycine max* samples and treatment

We used the fifth and sixth trifoliate *G. max* leaves in the analyses. Samples were separated immediately after leaf collection into two groups. The first part was sanitized with water, dried with paper towels, and kept in laboratory for biological assays with *S. cosmioides* caterpillars. The other parts were frozen with liquid nitrogen, transported to a chemical laboratory, partitioned into two samples, and stored. The first sample was lyophilized (Lyophilizer E-C Modulyo, Thermo Fisher, Scientific, Massachusetts, United States), milled, and subsequently fractioned using a mesh sieve of 80 mesh, and stored at −8°C. The second sample of this fresh material was stored using an ultra-freezer Sanyo MDF-U56VC (Panasonic, Osaka, Japan) at −80°C.

### *Spodoptera cosmioides* insect feeding experiment

Multiple and no-choice leaf consumption assays were adapted from the procedure described by Freitas et al. (2018). For the multiple-choice assay, a third instar caterpillar of *S. cosmioides* was placed in the center of an arena containing leaves of each cultivar. For the experiments with no-choice, there were leaves of only one cultivar in each compartment. After 60 h from the beginning of each experiment, consumed leaf area was determined using the LI-COR area meter (LICOR, Lincoln, NE, EUA). Each bioassay was composed of 10 replicates (*n* = 10). We used one arena for each repetition.

### Secondary metabolite extraction protocols for *Glycine max* leaves

We used a sample containing a mixture of leaves from all cultivars in order to optimize extraction methods. The responses (dependent variables) considered for measuring the quality of extraction methods were: the number of isolated compounds in the extracts and the sum of peak areas. The independent variables were selected based on previous studies conducted by our research group and on the studies from Liu et al. (2010), Silva et al. (2007), Song et al. (2014), and Zhang and Guo (2017). Details on the extraction methods are presented below.

### Nonvolatile organic compounds from *Glycine max* leaves

Since different parameters may affect the efficiency of solid–liquid extractions, a full-factorial design was performed in the first step as to determine which variables presented significant effects. Thus, a response surface methodology based on the central composite design was used for optimizing variable levels and ultimately improving extraction efficiency (Bezerra et al., 2008).

For conventional solid–liquid extraction (SLE), a full 2^4^ factorial design for evaluating temperature (25°C, 50°C, and 75°C) was performed. Extraction mixture – methanol solution (50%, 70%, and 90%), time (5, 20, and 35 min), and the number of extractions (1, 2, and 3) was also evaluated. For the experiments, lyophilized plant material (25 mg) was submitted to extraction within 1,000 μl of extraction mixture in a glass tube (8 ml) using a dry bath incubator with 700 rpm agitation (K80 Kasvi, Paraná, Brazil).

For the solid–liquid extraction assisted by ultrasound bath (UAE), three independent variables were evaluated: time (5, 20, and 35 min), extraction mixture – methanol solution (50%, 70%, and 90%), and the number of extractions (1, 2, and 3) in a 2^3^ full factorial design Thus, the lyophilized botanical material (25 mg) was submitted to extraction within 1,000 μl of extraction mixture in a glass tube (8 ml) in a 40 kHz ultrasound bath (USC 1400 Unique, São Paulo, Brazil).

After initial screening, a central 2^2^ composite experiment with two axial points was idealized in order to fully explore the remaining two variables with greater relevance from each extraction. Thus, we evaluated temperature (60°C, 70°C, 80°C, 90°C, and 100°C), and the number of extractions (3, 4, 5, 6, and 7) for SLE. Meanwhile, for UAE a water/methanol extraction mixture (40:60, 65:35, 70:30, 85:15, and 100:0% v/v), and number of extractions (3, 4, 5, 6, and 7) were evaluated.

In both extraction protocols, extracts were centrifuged (3,200 × *g*, for 1 min at 10°C), and the supernatant was collected, dried, and dissolved in 1.0 ml of the extraction mixture. For experiments with more than one extraction, the solvent was replaced after each cycle. After the last extraction cycle, the sum of all supernatants was dried and dissolved in 1,000 μl of the extraction mixture. Subsequently, the extracts were filtered in a 0.22 μm PTFE membrane, and 10 μl of the extracts were diluted in water for a final volume of 1,000 μl. The internal standard was added (10 μl of caffeine 1.0 μg⋅mL^−1^), and samples were analyzed by UHPLC-q-TOF-MS/MS (Agilent, 6,545 LC/Q-TOF).

### Volatile organic compounds from *Glycine max* leaves

For determining the optimal condition for volatile metabolites extraction by static headspace, a full 2^3^ factorial design and three central points were used. For this, three independent variables were evaluated: time (5, 15, and 25 min), temperature (40°C, 80°C, and 120°C), and saturation with glycerol (0, 0.5, and 1.0 g).

The fresh botanical material (100 mg) was submitted to headspace extraction using a 20 ml vial. In some experiments glycerol was added. Menthol was used as the internal standard by adding a 10 μl (500 μg⋅ml^−1^, solution prepared in glycerol) to the headspace vial. Vials were sealed with an aluminum cap and PTFE/SIL septum, and after volatile compound extraction, analyzed using a GC–MS (Shimadzu, GC–MS TQ-8040).

### Chromatographic analyses

The non-target analysis of the nonvolatile organic components was performed in an Ultra-High-Performance Liquid Chromatography system (Agilent 1290, Agilent Technologies, CA, United States) equipped with a Phenyl-hexyl Zorbax RRHD Eclipse Plus (2.1 × 100 mm, 1.8 μm Agilent) column as stationary phase. The column oven and autosampler temperatures were 45°C and 10°C, respectively. A constant flow gradient (0.35 ml⋅min^−1^) combining solvent A (0.1% formic acid/water) and solvent B (0.1% formic acid/methanol) was used under the following conditions: 8%–90% B (0–17 min) and 90% B (17–20 min) with 4 min of post-run. The injection volume was 1.0 μl. Mass spectrometry (MS) detection was performed through a Quadruple-Time-of-Flight Mass Spectrometry (Agilent 6,545 Q-TOF MS) equipped with an electrospray ionization source (ESI) in positive ion mode and capillary voltage of 3.5 kV. Source and desolvation gas temperatures were 350°C. Desolvation gas flow was 11 l⋅min^−1^, and gas flow in the cone was 8 l⋅min^−1^, with a fragment of 250 V, skimmer voltage of 65 V, and nozzle of 1,000 V. MS data were collected and ranged from 100 to 1,000 Da.

Volatile organic compounds were analyzed with a Shimadzu Gas Chromatography (GC 2010 Plus) coupled to a sequential mass spectrometer (MS TQ-8040) with a ZB-Wax capillary column (Phenomenex, stationary phase of polyethylene glycol, 30 m × 0.25 mm *d.i.* × 0.25 μm film thickness) and Helium 5.0 as a carrier gas (1 ml⋅min^−1^). Gas chromatographic parameters were as follows: 220°C injector temperature; the column temperature initially at 40°C maintained for 5 min, then heated to a rate of 8°C⋅min^−1^ to 240°C keeping at this temperature for 3 min. The interface and ion source temperatures were 250°C and 280°C, respectively. The injection was split mode (15:1), and the injected sample volume was 1,000 μl. The quadrupolar mass analyzer operated with electron ionization at 70 eV and scanning ranging from 40 to 500 Da.

Quality control samples (extracts containing the mixture of all cultivars) and internal standard solution were also continuously injected to monitor the stability of the systems.

### Data processing methods

The MS data obtained by liquid chromatography experiments were deconvoluted and integrated using the “Molecular Feature Extraction” algorithm in the Mass Hunter Qualitative Analysis Workflows B.08.00 Software (Agilent Technologies, CA, United States) and converted to a “.CSV” format. The identification of compounds was performed by comparison with METLIN, MassBank, and m/zcloudspectral data. For the multivariate analysis of the data from the gas chromatography system, raw data were converted into “.CSV” format. The identification of the compounds was performed by comparison with the spectral data from the NIST library (version 17.0) and with the AMDIS GC/MS software (version 2.73). The data for each treatment were combined and analyzed, then arranged into a matrix consisting of variables (columns), such as the relative peak abundance of each identified constituent, while objects (rows) were the sampled cultivars. The matrix was scaled, and then a PCA and HCA were performed using the MetaboAnalyst platform (version 4.0; Xia and Wishart, 2011).

### Statistical analyses

The different area and number values from compounds obtained through extraction optimization methods were evaluated through an analysis of variance (ANOVA), *F* test (5% probability), and determination of regression coefficients. These analyses were performed in order to verify the existence of significant differences between the studied variables in the extraction optimization conditions. The computational routines proposed by Pereira and Pereira-Filho (2018) and the Octave 4.4 software (2018) were used for these analyses.

In order to evaluate the data obtained in leaf consumption experiments with th*e S. cosmiodes* caterpillars, ANOVA and Tukey test (5% probability) were also used. The analyses were performed through the SAS^®^ statistical software version 9.4 58.

## Results and discussion

In the first stage of this study, we investigated solid–liquid extraction (SLE), solid–liquid extraction assisted by ultrasound bath (ultrasound-assisted extraction – UAE), and static headspace extraction techniques. We aimed to improve the analyses of different metabolites used as defense mechanisms against *S. cosmioides* caterpillars which are naturally derived from the phenotypic diversity in *G. max* populations. This step is fundamental for metabolomic analysis, ensuring a nearly full chemical profiling for comparison. Results from the development of extraction methodologies are described below.

### Nonvolatile organic compounds from *Glycine max* leaves

SLE is one of the conventional methods for sample preparation. This method is associated with the ultrasound process in order to accelerate mass transfer between a sample and the extraction mixture. It does so by forming, growing, and collapsing bubbles in the extractor solvent (cavitation; Vinatoru, 2001). As such, we evaluated SLE and UAE in order to verify the influence of these processes on extraction.

**Figure 1:**
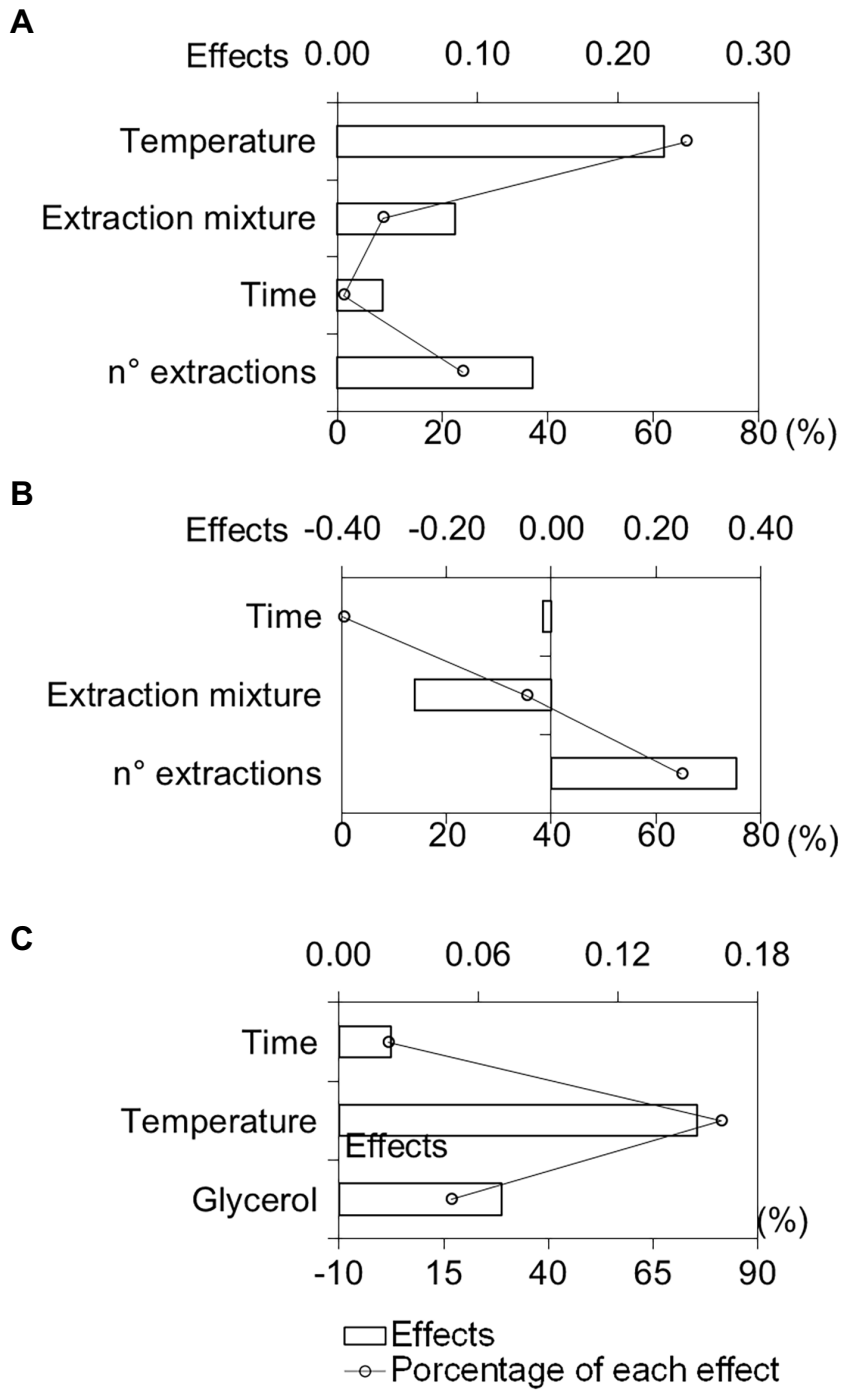
Graphic representation of the main differences in the independent variables and the percentage of each effect using solid–liquid extraction **(A)**, solid–liquid extraction assisted by ultrasound bath **(B)**, and headspace extraction **(C)**.

We calculated the effects of each independent variable based on the number and area of the compounds obtained after extraction (Supplementary Tables 1, 2). Response values were transformed into a dimensional weight named individual desirability (d*_i_*). Global desirability (D*_g_*) was derived from calculating the geometric mean d*_i_* values from each experiment (Lazic, 2004). The effect plots were constructed from the D*_g_* data, as shown in Figure 1A. Effects higher than 10% were considered significant.

Using SLE, we observed that temperature (A) and the number of extractions (D) showed the most significant influence in the process (Figure 1A).

The positive effect of temperature (Figure 1A) indicates better extraction of the organic compounds at higher temperatures. Similar results were obtained by Silva et al. (2007) and Liu et al. (2010), where temperatures above 90°C increased the yield in phenolic compound extraction from *Inga edulis* and *Gynura medica* leaves, respectively. The increase in temperature may favor the diffusion process of the matrix compounds to the solvent. Therefore, temperature helps rupture leaf cell wall components, promoting growth in diffusion coefficients, with a consequent increase in the solubility of chemical constituents (efficiency of extraction; Liu et al., 2010).

The evaluation of effects also revealed that the number of consecutive extractions had an important effect on efficiency (Figure 1A). This variable also favors the extraction of organic compounds by UAE (Figure 1B) and had a significant influence on extraction. Mosca et al. (2018) showed that the mass transfer process during SLE and UAE stops when the partition balance between the liquid and solid phase is reached. The renovation of extractor solvent in the multiple extractions leads to a return to initial equilibrium conditions and favors partitioning the extractable molecules from matrix to solvent. Thus, multiple extractions can provide a complete extraction of the chemical compounds from the matrix (Mosca et al., 2018). Cubas et al. (2008) observed similar effects on chlorophyll extraction in green beans. It is interesting to highlight that the increase in the number of extractions favored the extraction of phenolic compounds and derivatives (compounds with retention time longer than 5 min in the chromatographic analyses as can be seen in Supplementary Figure 1). The area sum for these compounds for ESL and UAE were 16.7 and 66.9%, respectively (Supplementary Tables 1, 2).

We verified that extraction mixture composition (B) also influenced the process in addition to the number of extractions in UAE. The negative effect of the extraction mixture (Figure 1B) indicated lower extraction of the compounds in higher organic solvent concentrations. Similar results were also obtained by Li et al. (2019) using ethanol proportions above 60% (v⋅v^−1^), due to a change in the polarity of the solvent which diminishes phenolic compound extraction.

After screening the variables and better informed on the extraction processes, we selected temperature, number of extractions, and extraction mixture compositions to be investigated in detail. A central 2^2^composite design with two axial points was performed for each extraction technique, and the analysis of variance from the data can be verified in Table 1.

**Table 1:** Variance analysis (95% confidence level).

Method	Source of variation	Sum of squares	Degrees of freedom	Mean squares	*F*	*F*_tab_
ESL	Regression	0.6786	5	0.1357	8.95	5.05
	Residual	0.0758	5	0.0152		
	Total	0.7544	10	0.0754		
	Pure error	0.0294	2	0.0147	1.05	19.16
	Lack of fit	0.0464	3	0.0155		
	*R^2^*	0.8995				
UAE	Regression	0.6298	5	0.1260	30.42	5.05
	Residual	0.0207	5	0.0041		
	Total	0.6505	10	0.0651		
	Pure error	0.0087	2	0.0044	0.91	19.16
	Lack of fit	0.0120	3	0.0040		
	*R^2^*	0.9682				

We obtained *R^2^* values of 0.8995 and 0.9682 for SLE and UAE extractions by central composite design, respectively. These values indicate that the models can explain at least 89% and 96% of the variability in the response. In addition to the explained variance, we evaluated the relationship between the *F*-calculated and tabled *F*-values. In this study, the regression relationship had values of 1.77 and 6.02 for ESL and UAE, respectively. *F*-calculated scores should be closer to 10-times larger than *F*-tabled in a regression in order to predict values (Box, 1978; Bezerra et al., 2008). The calculated *F*-value lacking adjustment was 0.055 and 0.048 for the ESL and UAE, respectively, indicating no lack of adjustment (ratio of <0.10). A contour surface plot was generated for each evaluated extraction method in order to portray the inclination of the processes (Supplementary Figures 2A,B).

The contour surface plots illustrate the relationship between extraction efficiency (given in desirability) and experimental variables. For SLE, we observed a linear increase in the extraction with desirability values above 0.70 with increases in temperature and number of extractions (Supplementary Figure 2A). For the UAE, we verified quadratic effects. The contour surface plots indicated that there is an increase in the extraction efficiency with increases in the number of extractions and a percentage of water between 40% and 60% (Supplementary Figure 2B).

When evaluating the results from these different techniques, we observed both to be efficient alternatives for extracting a wide variety of compounds (Supplementary Figure 1). However, ultrasound baths had the advantage of allowing the extraction of multiple samples simultaneously under the same operating conditions. Moreover, the optimal extraction parameters for UAE favored the extraction of phenolic compounds; a class previously pointed out as important for the discrimination of soybean cultivars (Supplementary Figure 1). Finally, high temperatures were not necessary in UAE, which can cause chemical degradations of organic compounds (Ghafoor et al., 2009).

Therefore, after optimization of the two methodologies, UAE was selected for extraction of metabolites from *G. max* leaves. Under the specified conditions, lyophilized plant material (25 mg) was subjected to extraction within 1,000 μl of an extraction mixture composed of water/methanol (50:50 v⋅v^−1^) in an ultrasound bath for 5 min. This process was carried out for six cycles.

### Volatile organic compounds from *Glycine max* leaves

The extraction of volatile compounds by static headspace is an automated technique that requires little to no sample preparation, decreasing the possibility of contamination, decomposition, and previous volatilization of molecules (Snow and Bullock, 2010). This technique also ensures that only the volatile molecules are introduced into the chromatographic injector (Soria et al., 2015), minimizing the number of steps and matrix effects compared to those traditionally used (e.g., solid–liquid extraction). With these advantages taken into consideration, the technique was evaluated in this study.

Through the results, we calculated the effects of each independent variable through the application of the desirability function (Supplementary Table 3) and obtained effect plots, as shown in Figure 1C.

Figure 1C shows that the time variable (A) presented a negligible effect (<10%). Only temperature (B) and glycerol saturation (C) influenced the extraction process (Figure 1C). We observed that temperature increase promoted a rise in compound intensity and quantity, representing a better extraction (positive effect). This result indicates that there is a vapor pressure increase in the sample at high temperatures, decreasing molecular matrix-analyte interactions. Consequently, a reduction in matrix analyte solubility decreases the partition coefficient, promoting a volatile fraction concentration increase in the headspace phase (Carvalho et al., 2007).

We also observed gains by adding glycerol (“solvent-assisted extraction”), which increased extraction efficiency and analytical sensitivity in the headspace analysis. Zhang and Guo (2017) demonstrated that internal and external wetting of the matrix is promoted by adding small amounts of a high boiling point solvent onto the solid matrix. Thus, with an increase in sample surface area exposure to extraction temperatures, there is a decrease in analyte solubility in the matrix and an increase in the vapor pressure, which improves the extraction efficiency of most volatiles contained in the solid sample (Zhang and Guo, 2017).

Given these results, we attempted to evaluate other variable levels that showed significant effects on the extraction process (temperature and saturation with glycerol). At temperatures above 120°C, however, there are signals of organic compound degradation within the headspace vial. We also recorded results from the addition of more than 1 g of glycerol in the samples, which presented a moisture accumulation within the headspace vial under heating, thus interfering with extraction efficiency. Thus, the extraction of volatiles was performed using 100 mg of fresh plant material, for 25 min at 120°C, and 1 g of glycerol. A chromatogram of the analysis obtained in the best extraction condition is illustrated in Supplementary Figure 3.

### Characterization of compounds extracted from *Glycine max* leaves

Nonvolatile and volatile organic compounds were extracted and evaluated from the leaves of all cultivars using the extraction protocols we developed. Once the extraction methods were selected, the composition of metabolites in the cultivars were evaluated individually. Total ion metabolite chromatograms are shown in Supplementary Figures 4, 5. Among the isolated compounds, only those with identified molecular formulas were selected for the multivariate analysis. Chemical information on the compounds is presented in Supplementary Tables 4, 5.

Phenolic glycosides were predominant among the nonvolatile organic compounds in *G. max* leaves from the ANTA 82 RR, CD 208, M8230 RR, P98Y11 RR, UFUS Capim Branco, and Xavante cultivars, corresponding to total relative area ranging from 45.5% to 64.4% (Table 2). When evaluating the identified compounds in these groups, we highlight kaempferol-3-*O*-*β*-D-glucopyranosyl(1 → 2)-*O*-[*α*-L-rhamnopyranosyl(1 → 6)]-*β*-D-galactopyranoside, kaempferol-3-*O*-[*α*-L-rhamnopyranosyl(1 → 6)]-*β*-D-glucopyranoside, and kaempferol-3-O-*α*-L-rhamnopyranosyl(1 → 6)-*β*-D-galactopyranoside since they present the largest relative areas in chromatograms (Table 2). These compounds, as well as kaempferol-3-O-*β*-D-(2,6-di-O-*α*-L-rhamnopyranosyl) galactopyranoside are associated with *G. max* leaves stage of development and are present in more significant amounts in young plants, such as the leaves in the vegetative stages as used in this study (Ho et al., 2002; Song et al., 2014). When accumulated in vacuoles or cellular walls, these compounds assist in leaf growth, formation, and pod morphology, as well as promoting tissue protection such as filtering UV-light (Song et al., 2014; Ishihara et al., 2016).

**Table 2:** *Glycine max* leaf nonvolatile compound relative areas extracted from 10 different cultivars.

PCA code	Compound	Relative area (mean ± SD)									
		ANTA 82 RR	CD 208	M8230 RR	P98Y11RR	PI 227682	UFUS Capim Branco	UFUS Carajas	UFUS Impacta	UFUS Milionaria	UFUS Xavante
AK	indole	0.13 ± 0.03	0.13 ± 0.01	0.75 ± 0.53	0.05 ± 0.01	0.68 ± 0.25	0.31 ± 0.03	0.03 ± 0.02	0.08 ± 0.04	0.57 ± 0.30	0.08 ± 0.01
AH	trigonelline	0.38 ± 0.07	0.78 ± 0.12	1.17 ± 0.06	0.74 ± 0.14	0.72 ± 0.07	1.60 ± 0.12	0.80 ± 0.19	1.01 ± 0.15	1.38 ± 0.50	0.66 ± 0.07
FA1	tuberonic acid glucoside	1.94 ± 0.44	3.84 ± 3.11	6.01 ± 1.13	1.87 ± 0.37	7.66 ± 0.57	7.56 ± 1.05	4.22 ± 0.49	2.38 ± 0.39	2.24 ± 0.18	1.85 ± 0.48
FA2	tetradecanoic acid	0.44 ± 0.28	0.46 ± 0.34	1.33 ± 0.40	1.61 ± 0.14	1.48 ± 0.11	2.69 ± 0.26	1.81 ± 0.23	1.21 ± 0.17	1.91 ± 0.04	0.71 ± 0.18
FA3	16-hydroxy hexadecanoic acid	0.73 ± 0.38	0.76 ± 0.42	0.53 ± 0.19	1.24 ± 0.42	1.23 ± 0.42	0.72 ± 0.36	1.37 ± 0.27	1.59 ± 0.48	2.40 ± 0.78	0.72 ± 0.20
FA4	palmitic amide	3.04 ± 0.40	3.34 ± 1.01	3.90 ± 0.37	6.95 ± 0.96	4.18 ± 1.82	5.53 ± 0.63	10.14 ± 0.93	10.76 ± 0.60	8.28 ± 1.10	3.61 ± 0.38
FE1	luteolin	0.39 ± 0.12	1.68 ± 0.10	0.35 ± 0.02	0.46 ± 0.04	nd	3.00 ± 0.79	0.39 ± 0.04	nd	nd	0.44 ± 0.08
FE2	3-*O*-methylquercetin	nd	nd	4.18 ± 0.23	nd	nd	2.42 ± 0.33	2.37 ± 0.27	nd	1.50 ± 0.24	nd
FE3	baicalein	0.52 ± 0.13	0.57 ± 0.03	0.02 ± 0.01	0.50 ± 0.08	0.03 ± 0.02	0.37 ± 0.52	0.02 ± 0.01	0.09 ± 0.05	0.07 ± 0.01	0.69 ± 0.21
FE4	apigenin	1.75 ± 0.38	2.38 ± 0.51	0.08 ± 0.02	0.87 ± 0.18	0.39 ± 0.51	0.17 ± 0.10	0.21 ± 0.16	0.17 ± 0.08	0.24 ± 0.06	1.26 ± 0.26
FE5	mosloflavone	1.44 ± 0.34	0.67 ± 0.33	3.08 ± 0.57	0.81 ± 0.16	6.63 ± 2.12	3.51 ± 0.77	1.44 ± 0.27	1.02 ± 0.57	3.22 ± 0.93	0.18 ± 0.04
FE6	chrysin	0.39 ± 0.12	0.14 ± 0.10	0.35 ± 0.14	0.11 ± 0.04	0.90 ± 0.24	1.23 ± 0.42	0.38 ± 0.32	0.52 ± 0.32	0.26 ± 0.23	0.13 ± 0.04
FLA	naringenin	0.11 ± 0.04	0.18 ± 0.09	0.08 ± 0.02	0.14 ± 0.03	0.09 ± 0.03	0.22 ± 0.07	0.13 ± 0.03	0.19 ± 0.04	0.24 ± 0.07	0.17 ± 0.07
IR	loganic acid	0.05 ± 0.01	0.02 ± 0.00	0.12 ± 0.01	0.02 ± 0.01	0.09 ± 0.09	0.11 ± 0.03	0.08 ± 0.01	0.08 ± 0.02	0.05 ± 0.00	0.23 ± 0.03
ISO1	glycitein	0.23 ± 0.03	0.27 ± 0.10	0.44 ± 0.08	0.12 ± 0.02	0.81 ± 0.26	0.65 ± 0.14	0.26 ± 0.15	0.24 ± 0.07	0.24 ± 0.12	0.08 ± 0.03
ISO2	6″-*O*-malonylgenistin	0.39 ± 0.09	0.21 ± 0.15	0.24 ± 0.04	0.10 ± 0.03	0.43 ± 0.16	0.88 ± 0.12	0.35 ± 0.21	0.26 ± 0.14	0.35 ± 0.24	0.27 ± 0.10
ISO3	daidzein	0.23 ± 0.05	0.09 ± 0.04	0.39 ± 0.14	0.25 ± 0.09	3.16 ± 0.21	1.21 ± 0.24	0.22 ± 0.09	0.98 ± 0.33	0.56 ± 0.28	0.10 ± 0.03
ISO4	biochanin A	0.05 ± 0.02	0.03 ± 0.02	0.11 ± 0.02	0.05 ± 0.01	0.39 ± 0.17	0.10 ± 0.01	0.07 ± 0.01	0.17 ± 0.06	0.20 ± 0.03	0.02 ± 0.00
ISO5	formononetin	3.55 ± 0.67	0.74 ± 0.10	2.13 ± 0.46	0.06 ± 0.02	1.53 ± 0.52	1.12 ± 0.21	0.52 ± 0.21	0.29 ± 0.11	0.60 ± 0.32	0.30 ± 0.04
ISO6	genistein	2.37 ± 0.38	4.82 ± 0.83	0.07 ± 0.02	1.18 ± 0.27	0.18 ± 0.13	0.21 ± 0.12	0.09 ± 0.05	0.28 ± 0.20	0.29 ± 0.14	1.55 ± 0.48
ISO7	afrormosin	3.95 ± 0.43	2.79 ± 0.18	8.31 ± 0.55	1.40 ± 0.32	19.20 ± 6.19	11.81 ± 2.22	6.38 ± 1.00	2.13 ± 0.44	5.50 ± 3.00	1.21 ± 0.20
JA	methyl jasmonate	0.08 ± 0.02	0.10 ± 0.01	0.25 ± 0.03	0.70 ± 0.09	0.61 ± 0.25	0.39 ± 0.03	0.94 ± 0.07	1.07 ± 0.10	0.19 ± 0.05	0.19 ± 0.02
OL	quercetin-3-*O*-glucosyl-rutinoside	nd	nd	0.34 ± 0.08	nd	0.02 ± 0.00	0.08 ± 0.01	1.09 ± 0.11	nd	0.26 ± 0.03	nd
PG1	methyl salicylate-*O*-[rhamnosyl-(1 → 6)-glucoside]	0.12 ± 0.02	0.62 ± 0.11	0.23 ± 0.07	0.04 ± 0.00	0.30 ± 0.01	0.33 ± 0.10	0.08 ± 0.03	0.08 ± 0.02	0.13 ± 0.02	0.14 ± 0.01
PG2	kaempferol-3-*O*-*β*-D-glucopyranosyl(1 → 2)-*O*-[*α*-L-rhamnopyranosyl(1 → 6)]-*β*-D-galactopyranoside	30.51 ± 1.46	0.28 ± 0.02	18.92 ± 1.91	29.46 ± 1.82	0.53 ± 0.22	1.36 ± 0.42	10.93 ± 0.93	0.66 ± 0.71	3.15 ± 0.57	32.76 ± 1.52
PG3	kaempferol-3-*O*-*β*-D-glucopyranosyl(1 → 2)-*O*-[*α*-L-rhamnopyranosyl(1 → 6)]-*β*-D-glucopyranoside	17.71 ± 1.41	0.15 ± 0.01	10.74 ± 1.11	12.13 ± 2.13	0.18 ± 0.14	1.64 ± 0.49	6.60 ± 1.26	0.53 ± 0.33	3.32 ± 0.09	18.87 ± 1.17
PG4	kaempferol-3-*O*-digalactopyranoside	3.20 ± 0.40	0.35 ± 0.03	3.73 ± 0.86	1.56 ± 0.33	0.07 ± 0.06	2.84 ± 1.82	0.95 ± 0.15	0.13 ± 0.08	0.42 ± 0.16	1.64 ± 0.28
PG5	kaempferol-3-*O*-diglucopyranoside	1.78 ± 0.26	0.19 ± 0.05	0.97 ± 0.20	1.02 ± 0.06	0.04 ± 0.03	2.12 ± 1.06	0.41 ± 0.02	0.08 ± 0.07	0.21 ± 0.10	1.05 ± 0.15
PG6	kaempferol-3-*O*-*β*-D-(2,6-di-*O*-*α*-L-rhamnopyranosyl)-galactopyranoside	3.67 ± 1.82	17.65 ± 3.16	4.72 ± 2.26	2.90 ± 0.54	0.04 ± 0.05	1.97 ± 0.53	1.96 ± 0.53	0.51 ± 0.15	1.26 ± 0.23	5.96 ± 1.33
PG7	isorhamnetin glycoside	nd	nd	nd	nd	nd	1.37 ± 0.15	nd	nd	nd	nd
PG8	isorhamnetin glycoside	nd	nd	0.08 ± 0.02	nd	nd	1.63 ± 0.38	0.01 ± 0.00	nd	nd	nd
PG9	kaempferol-3-*O*-*α*-L-rhamnopyranosyl(1 → 6)-*β*-D-galactopyranoside	1.29 ± 0.10	14.84 ± 1.13	1.78 ± 0.14	1.46 ± 0.27	5.96 ± 3.28	12.98 ± 3.18	1.62 ± 0.36	14.68 ± 2.11	11.96 ± 1.77	1.90 ± 0.18
PG10	astragalin	0.28 ± 0.05	0.29 ± 0.05	0.15 ± 0.03	0.18 ± 0.00	0.06 ± 0.04	2.71 ± 1.45	0.26 ± 0.34	0.06 ± 0.02	0.09 ± 0.02	0.15 ± 0.00
PG11	kaempferol-3-*O*-*α*-L-rhamnopyranosyl(1 → 6)-*β*-D-glucopyronoside	1.04 ± 0.14	20.30 ± 1.36	1.22 ± 0.12	1.11 ± 0.14	3.33 ± 1.72	4.25 ± 4.16	0.79 ± 0.15	3.47 ± 0.29	3.08 ± 1.06	1.62 ± 0.24
PG12	kaempferol glycoside	0.46 ± 0.09	0.30 ± 0.02	0.14 ± 0.02	0.12 ± 0.01	0.09 ± 0.05	3.71 ± 3.76	0.05 ± 0.02	0.10 ± 0.03	0.04 ± 0.01	0.31 ± 0.05
PG13	isorhamnetin glycoside	nd	nd	0.08 ± 0.02	nd	0.05 ± 0.03	1.10 ± 0.91	0.03 ± 0.01	0.04 ± 0.04	0.02 ± 0.01	nd
PG14	isorhamnetin glycoside	nd	0.01 ± 0.00	1.56 ± 0.37	nd	3.36 ± 1.60	3.29 ± 2.54	0.40 ± 0.10	1.65 ± 2.35	3.58 ± 2.00	0.01 ± 0.01
PG15	isorhamnetin glycoside	nd	0.01 ± 0.00	1.16 ± 0.26	nd	2.73 ± 1.25	2.49 ± 1.92	0.36 ± 0.07	1.73 ± 1.17	1.99 ± 0.40	0.01 ± 0.01
PG16	isorhamnetin glycoside	nd	nd	0.07 ± 0.01	nd	0.09 ± 0.04	3.56 ± 3.00	0.03 ± 0.01	0.07 ± 0.03	0.03 ± 0.01	nd
SB1	hexadecasphinganine	8.51 ± 0.78	9.23 ± 1.38	9.68 ± 0.76	18.77 ± 3.28	24.87 ± 2.04	18.51 ± 2.51	25.28 ± 0.79	26.52 ± 1.37	27.54 ± 2.23	9.32 ± 0.90
SB2	phytosphingosine	7.08 ± 1.56	7.50 ± 2.15	8.89 ± 0.94	13.55 ± 1.57	23.48 ± 2.18	12.84 ± 1.38	20.17 ± 1.84	22.60 ± 0.64	22.20 ± 2.23	8.59 ± 1.00
UN	sesquiterpene	0.46 ± 0.09	0.54 ± 0.03	0.52 ± 0.02	0.32 ± 0.05	0.57 ± 0.22	0.71 ± 0.14	0.40 ± 0.05	0.46 ± 0.04	0.94 ± 0.05	0.31 ± 0.02

SD, standard deviation; AK, alkaloid; AH, aromatic heterocyclic; FA, fatty acyl; FE, flavone; FLA, flavanone; IR, iridoid; ISO, isoflavone; JA, jasmonate; OL, oligosaccharides; PG, phenolic glycosides; SB, sphingoid base; UN, unidentified; nd, non-detected.

Lipids containing sphingoid bases were also detected as the class of molecules with the highest abundance for genotypes PI 227682, UFUS: Carajás, Impacta, and Milionária, presenting a total relative area of 45.45%–49.74% (Table 2). Plants can use these compounds as structural molecules, cell signals, and secondary messengers in order to regulate responses to stress, apoptosis, etc. (Sperling and Heinz, 2003; Berkey et al., 2012).

Other nonvolatile compounds detected in greater diversity were flavonoids, in particular isoflavones, representing 3.2%–25.7% of the total relative area in the chromatograms (Table 2). The major compounds in this group, genistein, and afrormosin, have already been detected in *G. max* leaves and seeds (Caballero et al., 1986; Ho et al., 2002; Gomez et al., 2018). These compounds are constitutively produced by soybean plants and perform an important role in adapting to the environment, as they may act as mediators in stressful environments and herbivory (Gomez et al., 2018). In addition to the PI 227682 cultivar, genistein and afrormosin were the second class of molecules with the highest abundances (25.7% of the total relative area; Table 2).

In regards to *G. max* volatile organic compounds from leaves, aldehydes, alcohols, and aromatic heterocyclic represented more than 80% of the total relative area in the chromatograms for all evaluated cultivars (Table 3). Aldehydes were the major compounds ranging from 30.4% to 38.0% of the total relative area for six of the 10 evaluated cultivars (ANTA 82 RR, CD 208, M8230 RR, PI 227682, UFUS: Milionária and Xavante). The main aldehyde was 3-methyl-butanal, with levels ranging from 9.2% to 16.2% (Table 3). In addition to that, we identified the following hexanals: hex-3-enal, *trans*-hex-2-enal, and *trans*,*trans*-hex-2,4-dienal as green leaf volatiles (GLVs) that contribute to the typical aroma of leaves (Holopainen and Gershenzon, 2010).

**Table 3:** Volatile compoundrelative areas from *Glycine max* leaf extract from 10 cultivars.

PCA code	Compound	Relative area (mean ± SD)									
		ANTA 82 RR	CD 208	M8230 RR	P98Y11 RR	PI 227682	UFUS Capim Branco	UFUS Carajas	UFUS Impacta	UFUS Milionaria	UFUS Xavante
A1	acetaldehyde	6.70 ± 0.51	8.02 ± 0.56	6.65 ± 0.40	7.68 ± 0.57	7.76 ± 1.07	7.46 ± 0.13	6.30 ± 0.53	7.50 ± 0.24	8.80 ± 0.89	8.19 ± 0.68
A2	propanal	0.68 ± 0.10	0.64 ± 0.06	0.77 ± 0.04	0.70 ± 0.05	0.59 ± 0.08	0.80 ± 0.09	0.59 ± 0.10	0.74 ± 0.10	0.76 ± 0.13	0.81 ± 0.07
A3	2-propenal	0.75 ± 0.12	0.53 ± 0.13	0.34 ± 0.09	0.27 ± 0.04	0.46 ± 0.12	0.36 ± 0.10	0.85 ± 0.13	0.58 ± 0.08	0.60 ± 0.12	0.86 ± 0.07
A4	butanal	0.51 ± 0.07	0.47 ± 0.15	0.52 ± 0.10	0.60 ± 0.19	0.45 ± 0.12	0.47 ± 0.08	0.45 ± 0.12	0.44 ± 0.03	0.53 ± 0.07	0.50 ± 0.02
A5	2-methyl-butanal	0.32 ± 0.01	0.28 ± 0.02	0.25 ± 0.08	0.24 ± 0.09	0.44 ± 0.19	0.31 ± 0.08	0.11 ± 0.01	0.20 ± 0.01	0.39 ± 0.23	0.24 ± 0.02
A6	3-methyl-butanal	12.61 ± 1.40	13.12 ± 1.91	9.17 ± 0.35	16.59 ± 0.49	10.69 ± 0.89	10.71 ± 0.16	7.09 ± 0.67	8.70 ± 0.59	16.24 ± 0.63	10.71 ± 0.49
A7	hexanal	5.98 ± 0.91	5.71 ± 0.33	9.05 ± 5.30	3.45 ± 0.33	3.33 ± 0.14	4.98 ± 0.51	3.40 ± 0.33	3.56 ± 0.32	3.84 ± 0.13	6.24 ± 2.70
A8	trans-2-pentenal	0.49 ± 0.09	0.40 ± 0.16	0.54 ± 0.18	0.39 ± 0.09	0.35 ± 0.12	0.46 ± 0.07	0.47 ± 0.06	0.44 ± 0.04	0.51 ± 0.05	0.34 ± 0.04
A9	hex-3-enal	0.29 ± 0.05	0.32 ± 0.08	0.37 ± 0.06	0.25 ± 0.07	0.21 ± 0.03	0.23 ± 0.01	0.24 ± 0.04	0.26 ± 0.05	0.25 ± 0.04	0.41 ± 0.14
A10	pentanal	nd	nd	nd	nd	nd	0.27 ± 0.01	nd	nd	nd	nd
A11	trans-hex-2-enal	8.13 ± 1.41	6.03 ± 0.72	9.27 ± 3.34	4.60 ± 1.05	4.70 ± 0.25	5.37 ± 0.53	4.76 ± 0.62	5.15 ± 0.25	5.22 ± 0.78	8.02 ± 2.68
A12	octanal	0.08 ± 0.00	nd	nd	0.08 ± 0.01	0.16 ± 0.01	0.08 ± 0.01	nd	nd	nd	nd
A13	nonanal	0.32 ± 0.03	0.19 ± 0.09	0.15 ± 0.02	0.12 ± 0.05	0.37 ± 0.05	0.17 ± 0.07	0.15 ± 0.04	0.11 ± 0.10	0.17 ± 0.01	0.15 ± 0.02
A14	trans,trans-hexa-2,4-dienal	0.24 ± 0.07	0.18 ± 0.01	0.21 ± 0.04	0.18 ± 0.02	0.21 ± 0.01	0.17 ± 0.05	0.18 ± 0.02	0.19 ± 0.07	0.15 ± 0.03	0.19 ± 0.03
A15	trans-2-trans-4-heptadienal	0.17 ± 0.04	0.16 ± 0.02	0.12 ± 0.01	0.14 ± 0.02	0.18 ± 0.02	0.18 ± 0.01	0.22 ± 0.04	0.15 ± 0.04	0.13 ± 0.03	0.17 ± 0.02
A16	heptadecanal	0.60 ± 0.03	0.10 ± 0.01	0.42 ± 0.05	0.18 ± 0.05	0.51 ± 0.10	0.26 ± 0.02	0.20 ± 0.02	0.15 ± 0.01	0.35 ± 0.16	nd
AL1	1-penten-3-ol	2.27 ± 0.13	1.69 ± 0.17	2.43 ± 0.18	1.61 ± 0.12	2.65 ± 0.19	1.89 ± 0.05	1.43 ± 0.25	1.72 ± 0.11	2.29 ± 0.28	2.56 ± 0.22
AL2	1-pentanol	0.13 ± 0.03	0.11 ± 0.03	0.15 ± 0.01	0.09 ± 0.02	0.18 ± 0.02	0.15 ± 0.03	0.08 ± 0.01	0.19 ± 0.06	0.19 ± 0.01	0.22 ± 0.03
AL3	cis-2-penten-1-ol	0.97 ± 0.18	0.86 ± 0.04	1.22 ± 0.13	0.88 ± 0.19	0.93 ± 0.08	0.78 ± 0.22	0.71 ± 0.12	0.71 ± 0.13	1.13 ± 0.11	1.55 ± 0.30
AL4	1-hexanol	0.51 ± 0.20	0.56 ± 0.24	0.34 ± 0.06	0.92 ± 0.21	2.27 ± 0.10	0.30 ± 0.09	2.03 ± 0.20	2.14 ± 0.15	0.91 ± 0.19	1.52 ± 0.14
AL5	trans-3-hexen-1-ol	0.38 ± 0.06	0.38 ± 0.09	0.49 ± 0.08	0.28 ± 0.07	0.67 ± 0.14	0.25 ± 0.03	0.81 ± 0.16	0.69 ± 0.18	0.56 ± 0.04	0.68 ± 0.04
AL6	trans-2-hexen-1-ol	0.35 ± 0.19	0.66 ± 0.08	0.17 ± 0.07	0.23 ± 0.08	0.70 ± 0.18	0.18 ± 0.07	1.01 ± 0.04	0.57 ± 0.06	0.81 ± 0.16	1.43 ± 0.15
AL7	1-octen-3-ol	15.48 ± 0.46	19.63 ± 1.66	23.97 ± 1.64	27.87 ± 2.07	19.74 ± 1.23	15.86 ± 0.54	31.31 ± 1.70	18.26 ± 1.43	21.30 ± 0.28	20.34 ± 1.08
AL8	1-octanol	0.20 ± 0.03	0.16 ± 0.01	0.17 ± 0.04	0.22 ± 0.07	0.18 ± 0.01	0.17 ± 0.04	0.29 ± 0.07	0.13 ± 0.05	0.16 ± 0.05	0.23 ± 0.06
AL9	3,4-dimethylcyclohexanol	0.21 ± 0.01	0.16 ± 0.01	0.16 ± 0.02	0.22 ± 0.05	0.22 ± 0.02	0.21 ± 0.02	0.23 ± 0.01	0.23 ± 0.11	0.33 ± 0.06	0.17 ± 0.05
H1	3,5,5-trimethyl-hex-2-ene	0.30 ± 0.02	0.23 ± 0.07	0.28 ± 0.05	0.27 ± 0.02	0.29 ± 0.01	0.24 ± 0.02	0.41 ± 0.06	0.24 ± 0.01	0.35 ± 0.02	0.25 ± 0.01
HA1	2-ethylfuran	29.34 ± 0.78	24.49 ± 1.88	32.61 ± 1.65	41.70 ± 0.27	22.68 ± 1.32	35.35 ± 0.15	17.11 ± 1.74	33.08 ± 1.51	32.76 ± 2.83	26.34 ± 0.42
HA2	2-pentyl-furan	0.29 ± 0.01	0.38 ± 0.06	0.28 ± 0.05	0.32 ± 0.07	0.45 ± 0.08	0.29 ± 0.03	0.31 ± 0.08	0.45 ± 0.04	0.29 ± 0.06	0.51 ± 0.08
HA3	furfural	0.22 ± 0.02	0.25 ± 0.05	0.19 ± 0.05	0.21 ± 0.04	0.23 ± 0.04	0.28 ± 0.04	0.28 ± 0.02	0.23 ± 0.02	0.30 ± 0.02	0.28 ± 0.03
K1	2-butanone	0.31 ± 0.07	0.45 ± 0.10	0.48 ± 0.02	0.36 ± 0,07	0.51 ± 0.13	0.44 ± 0.04	0.65 ± 0.07	0.48 ± 0.08	0.88 ± 0.05	0.47 ± 0.02
K2	3-methyl-2-butanone	nd	nd	nd	nd	nd	nd	0.25 ± 0.02	nd	nd	nd
K3	2,3-butanedione	5.40 ± 0.22	6.22 ± 0.70	4.11 ± 0.38	5.29 ± 1.33	6.10 ± 0.17	4.57 ± 0.35	4.99 ± 0.60	4.76 ± 0.33	5.89 ± 1.05	6.06 ± 0.80
K4	2,3-pentanedione	nd	5.25 ± 0.12	0.16 ± 0.02	0.15 ± 0.03	0.12 ± 0.02	0.25 ± 0.05	nd	nd	0.26 ± 0.02	0.18 ± 0.03
K5	3-octanone	0.14 ± 0.08	0.09 ± 0.02		0.18 ± 0.03	0.29 ± 0.09	0.16 ± 0.05	0.44 ± 0.10	0.19 ± 0.03	0.16 ± 0.00	0.12 ± 0.04
K6	acetoin	0.13 ± 0.01	nd	nd	0.23 ± 0.30	nd	nd	nd	nd	nd	nd
OC1	D-limonene	0.22 ± 0.09	0.26 ± 0.08	0.31 ± 0.16	0.44 ± 0.09	0.42 ± 0.09	0.46 ± 0.20	0.61 ± 0.18	0.51 ± 0.05	0.24 ± 0.09	0.30 ± 0.08
OC2	*β*-cymene	nd	nd	nd	nd	nd	nd	nd	nd	0.14 ± 0.01	nd
OC3	acetol	0.18 ± 0.03	0.08 ± 0.01	0.08 ± 0.00	0.05 ± 0.02	0.06 ± 0.00	0.11 ± 0.06	0.13 ± 0.01	0.16 ± 0.05	1.12 ± 0.06	0.13 ± 0.10
OC4	benzaldehyde	0.14 ± 0.05	0.84 ± 0.07	0.84 ± 0.04	0.90 ± 0.20	0.84 ± 0.17	0.79 ± 0.04	0.61 ± 0.10	0.65 ± 0.17	1.78 ± 0.27	0.83 ± 0.04
OC5	linalool	0.59 ± 0.14	1.04 ± 0.41	0.99 ± 0.17	0.35 ± 0.02	0.68 ± 0.14	0.80 ± 0.20	0.53 ± 0.15	0.51 ± 0.17	0.63 ± 0.29	0.47 ± 0.15
OC6	1,3,4-trimethyl-cyclohexene-1-carboxaldehyde	0.46 ± 0.10	0.42 ± 0.07	0.54 ± 0.01	0.52 ± 0.04	0.46 ± 0.03	0.34 ± 0.27	0.44 ± 0.22	0.43 ± 0.13	0.51 ± 0.01	0.59 ± 0.08
OC7	benzeneacetaldehyde	0.08 ± 0.01	0.08 ± 0.03	0.10 ± 0.02	0.07 ± 0.02	0.16 ± 0.04	0.10 ± 0.01	0.10 ± 0.01	0.09 ± 0.01	0.19 ± 0.07	0.09 ± 0.02
OC8	methyl salicylate	nd	nd	nd	nd	0.21 ± 0.01	nd	nd	nd	nd	nd
OC9	6,10-dimethyl-5,9-undecadien-2-one	0.28 ± 0.12	0.13 ± 0.04	0.13 ± 0.01	0.14 ± 0.02	0.27 ± 0.03	0.17 ± 0.05	0.22 ± 0.03	0.24 ± 0.13	0.18 ± 0.02	0.25 ± 0.02
OC10	*β*-ionone	0.53 ± 0.03	0.44 ± 0.03	0.63 ± 0.01	0.62 ± 0.06	0.58 ± 0.08	0.66 ± 0.13	0.54 ± 0.08	0.56 ± 0.10	0.72 ± 0.10	0.68 ± 0.08
OC11	4-vinylguaiacol	0.28 ± 0.03	0.30 ± 0.05	0.19 ± 0.06	0.21 ± 0.09	0.26 ± 0.02	0.18 ± 0.04	0.23 ± 0.04	0.28 ± 0.12	0.25 ± 0.04	0.16 ± 0.08
OC12	phytol	0.21 ± 0.07	0.18 ± 0.07	0.19 ± 0.01	0.29 ± 0.03	0.45 ± 0.07	0.24 ± 0.16	0.37 ± 0.06	nd	0.25 ± 0.07	0.53 ± 0.10

SD, standard deviation; A, aldehyde; K, ketone; HA, heterocyclic aromatic; OC, organic compound; AL, alcohol; H, hydrocarbon; nd, non-detected.

The relative area for alcohols in the chromatograms ranged from 19.8% to 37.9%, and we highlight 1-hexanol, *trans*-3-hexenol, and *trans*-2-hexen-1-ol. These compounds have also been classified as GLVs and oxygenated molecules. Among these compounds, 1-hexanol is considered a significant chemical in plant defense of damaged tissues. Among the alcohols, 1-octen-3-ol was the major compound present between 15.5% and 31.3% of the total relative area in the chromatograms. It was the main alcohol molecule in volatile fractions and the compound with highest relative abundance in the UFUS Carajás cultivar. In the studies conducted by Lee and Shibamoto (2000), this was the major component in the volatile composition of soybean grains. Furthermore, Kishimoto et al. (2007) also demonstrated that this is a common component in cowpea leaves.

Among aromatic heterocyclic compounds (17.7%–42.2% of the total relative area), we observed that all identified molecules were derived from the furan ring. In this group, we can highlight 2-ethylfuran because it was the main molecule in the volatile fraction of ANTA 82 RR, CD 208, M8230 RR, P98Y11 RR, PI 227682, UFUS: Capim Branco, Impacta, Milionária, and Xavante genotypes (Table 3). These molecules have previously been identified in soybean and tomato leaves (Snyder and King, 1994; Wang et al., 2001). However, the mechanism of how furan-derived molecules are formed, or their function in plants is not yet well described.

We grouped terpenes, such as *β*-cymene and D-limonene, and other organic functions as non-oxygenated compounds. This group represented <7% of the total relative area in the chromatograms (Table 3).

We verified that the genotypes showed different chemical profiling among each other. These qualitative and quantitative differences are probably due to genetic differences or gene expression among soybean genotypes (Azeez et al., 2018). The environment and geography could not be related to the chemical profiling distinction among samples, since plants were submitted to the same agricultural practices, grown in the same area, and collected at the same age and date.

### Influence of chemical profile on the resistance of *Glycine max* cultivars

We performed biological assays in order to evaluate how *G. max* leaf metabolites could influence *S. cosmioides* caterpillars feeding behavior. The results are presented in Supplementary Figure 6.

Leaf consumption analysis showed that *S. cosmioides* caterpillars respond differentially to feeding treatments. Supplementary Figure 6 shows that PI227682, UFUS Carajás, UFUS Xavante, UFUS Milionária, UFUS Impacta, and M8230RR genotypes showed an average consumption below 4.00 cm^2^ in multiple-choice assays. In no-choice assays, the PI227682 and UFUS Carajás cultivars maintained the lowest leaf consumption (0.73 ± 0.14 and 2.12 ± 0.31 cm^2^, respectively), followed by UFUS Milionária (3.61 ± 0.56), and UFUS Impacta (3.02 ± 0.15). The P98Y11 RR, M8230 RR, and UFUS Capim Branco cultivars were more consumed, with leaf consumption above 5.00 cm^2^ (Supplementary Figure 6). With this data, PI227682 and UFUS Carajás cultivars were classified as highly resistant; UFUS: Milionária, Xavante, and Impacta as resistant; and P98Y11 RR, ANTA82 RR, CD208, M8230 RR, and UFUS Capim Branco as susceptible to *S. cosmioides*. These data are supported by Freitas et al. (2018), who also classified P98Y11 RR and PI 227682 as susceptible and resistant to *S. cosmioides*, respectively.

**Figure 2:**
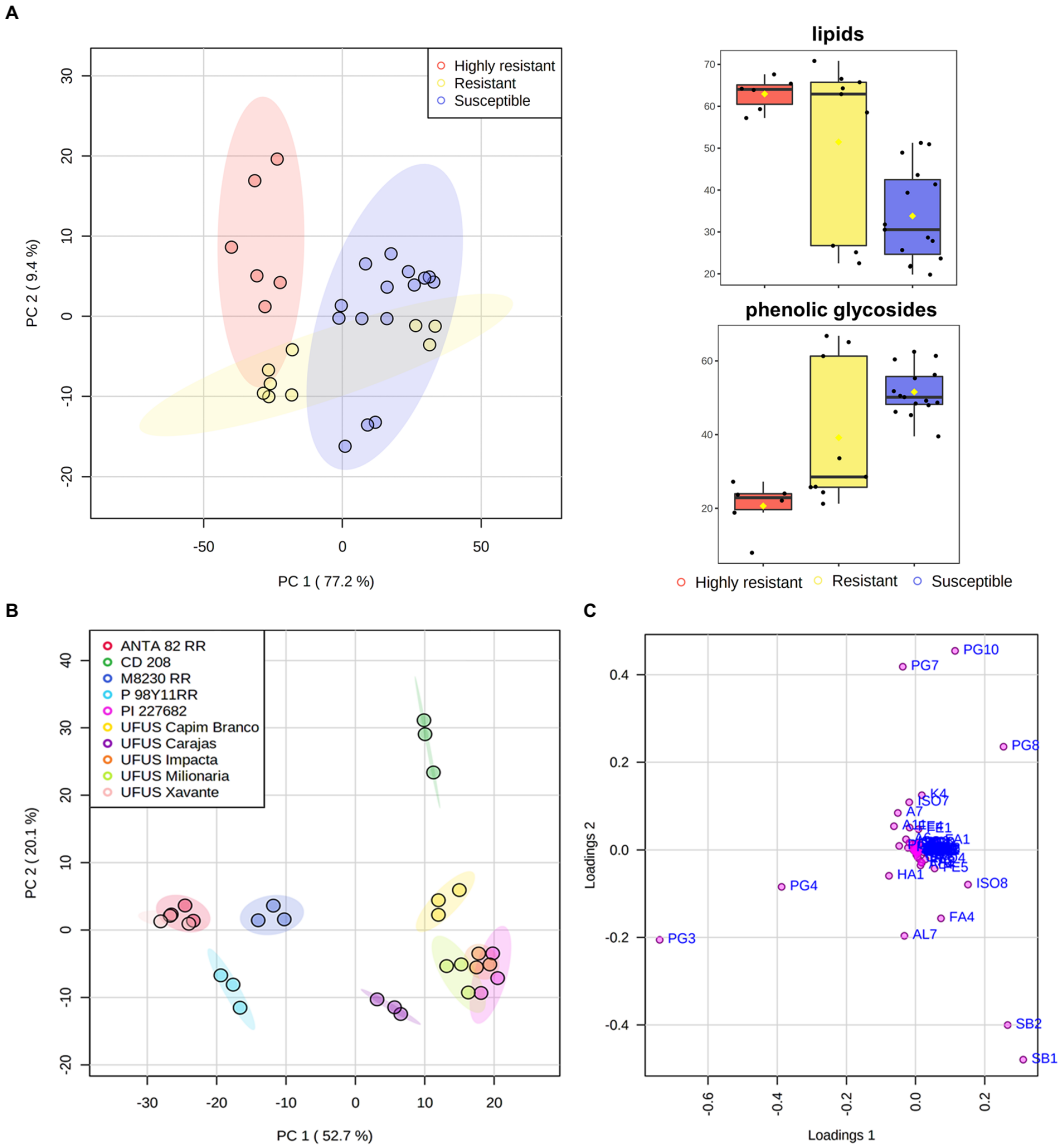
First and second component scores **(A, B)** and loadings **(C)** from samples and variables. These are arranged according to the influence of relative areas from nonvolatile and volatile compound chromatographic signals found in leaves of *Glycine max* (codes of the variables listed in Tables 2, 3). The color shaded area in each cluster represents the 95% confidence region. Box-and-whisker plots of lipids and phenolic glycosides relative area detected in *G. max* leaves. Y-axis represents the normalized sum of chromatographic peak area.

We performed a detailed multivariate analysis in order to investigate inclinations and data groupings, as well as to identify the main metabolites that affect leaf consumption by *S. cosmioides* caterpillars (Figure 2).

A clear separation between highly resistant and susceptible cultivars (Figure 2A) is present in the principal component analysis (PCA). The variation in the total content of lipids and glycosylated phenolic compounds may explain this difference (Figure 2). The influence of these compound classes is also observed and cultivars are individually evaluated in a PCA (Figure 2B). Figure 2C shows that the PI 227682, UFUS Impacta, Carajás, and Milionária leaf samples (resistant cultivars) are mainly influenced by lipids and the isoflavone afrormosin. Lipids with a sphingoid base can function as stabilizing structural cell membrane components. An increase of these compounds in the cell membrane may interfere with palatability and digestibility of *G. max* leaves (Huby et al., 2020), and thus, drive down leaf consumption by *S. cosmioides* caterpillars.

In Figure 2C, we observed that the P98Y11 RR, ANTA82 RR, CD208, and M8230 RR samples, which were classified as susceptible cultivars, were influenced by glycosylated phenolics derived from kaempferol, presenting a higher total content compared to resistant ones (Figure 2C). These compounds can also function as plant defense molecules, despite appearing to have been phagostimulants for *G. max* susceptible cultivars. Tahvanainen et al. (1985) reported that some specialized herbivore insects might use glycosylated phenolic compounds as a defense mechanism, therefore, they prefer to feed on leaves rich in these compounds. A similar process was observed in our study. Nevertheless, there are no detailed data on *S. cosmioides* behavioral responses (a generalist insect) to glycosylated kaempferol derivatives that prove this hypothesis.

Figure 2B shows that PC_1_ is responsible for 52.7% of the total variance and differentiated the ANTA 82 RR, M 8239 RR, P98Y11 RR, UFUS Xavante from CD 208, PI 227682, UFUS: Capim Branco, Carajás, Impacta, and Milionária cultivars. This allowed the grouping of the soybean cultivars by herbicide glyphosate tolerance transgene (or *Roundup Ready* – RR; Bøhn et al., 2014). Nevertheless, we cannot infer that metabolite variability can be a direct consequence of transgene insertion. We also have UFUS Xavante, a conventional cultivar obtained through genetic improvement in this grouping (Jorge et al., 2019).

**Figure 3:**
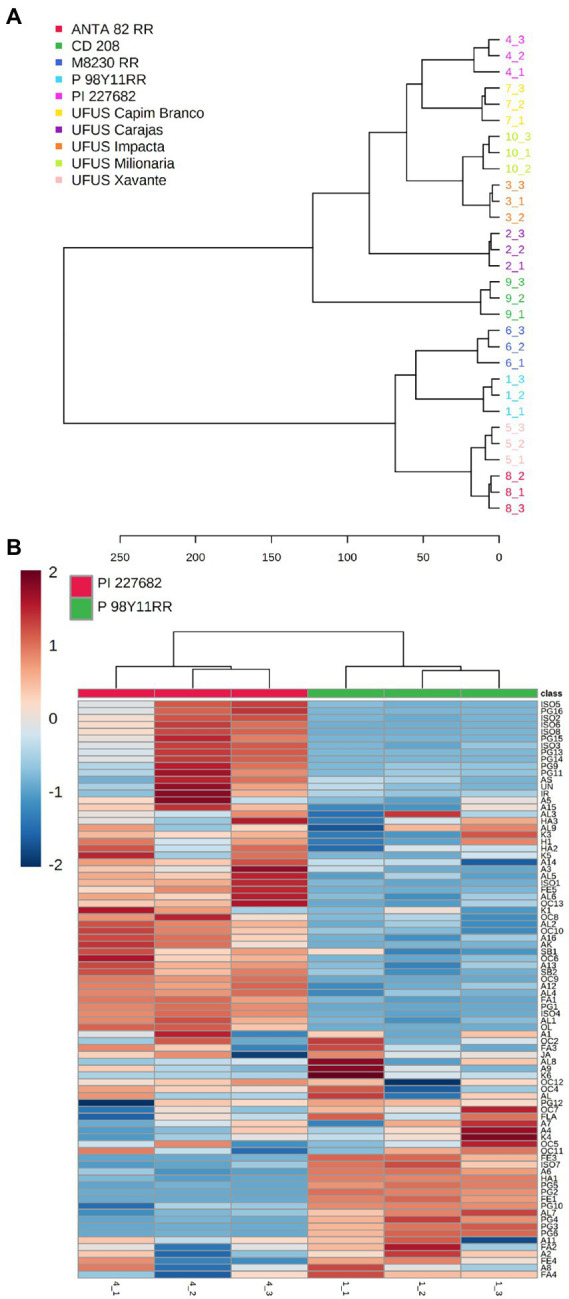
**(A)** Dendrogram showing the formation of groups according to similarity, indicating the similarity of the samples by distance, with groups formed from different *Glycine max* cultivars influenced by nonvolatile and volatile compounds. **(B)** Heatmap. Samples are represented in columns and metabolites represented by rows. The abundance of each metabolite is represented by a specific color. Upwards and downward regulated metabolites are indicated by different shades of red and blue, respectively.

Figure 3A shows similarities based on Euclidean distances of the samples. The resulting dendrogram shows the existence of two major clusters. In the first cluster, the cultivars ANTA 82 RR, and UFUS Xavante showed more significant similarities to M 8239 RR, and P98Y11 RR. The four cultivars formed a cluster, and the dendrogram in Figure 3A reflects the PC_1_ and PC_2_ scores (Figure 2B). The dendrogram also shows that the samples of conventional cultivars CD 208, PI 227682, and UFUS: Capim Branco, Carajás, Impacta, and Milionária are combined to form a larger cluster.

### The relationship between resistant/susceptible cultivars and metabolic pathways

We compared the metabolic profile of P98Y11 RR, a susceptible cultivar to *S. cosmioides*, to PI 227682, a highly resistant one as to obtain a detailed analysis of the critical metabolites for *G. max* defense mechanisms against *S. cosmioides* caterpillars. We use the *G. max* metabolic routes available in the Kyoto Encyclopedia of Genes and Genomes (KEGG).

We found that the resistant cultivar (PI 227682) presented a higher accumulation of various metabolites when compared to the susceptible cultivar (Figure 3B). The diversity in the chemical profiling may relate to the significant genetic variability in the susceptible cultivar (Berg, 2009; Azeez et al., 2018). Cultivar PI 227682 was obtained by selection and natural crossing, which tends to present more genetic variability (Berg, 2009; Azeez et al., 2018).

In Table 4, we present the compounds described in Figure 3A showing differences in relative abundance with *p* < 0.05 for *t*-test and fold change ≥2. A careful examination of the compounds allowed us to verify that the resistant cultivar also presented the highest accumulation of metabolites.

**Table 4:** Differentially expressed metabolites in the resistant cultivar with *p* < 0.05 for *t*-test and fold change ≥2 and relative abundances.

Compound	Fold change	Regulation	log2 (Fold Change)
*aldehyde*			
octanal	2.06E+00	Up	1.04
nonanal	3.00E+00	Up	1.59
heptadecanal	2.75E+00	Up	1.46
*alkaloid*			
indole	1.36E+01	Up	3.76
*alcohol*			
1-hexanol	2.46E+00	Up	1.29
trans-3-hexen-1-ol	2.38E+00	Up	1.25
trans-2-hexen-1-ol	3.00E+00	Up	1.58
*fatty acyl*			
tuberonic acid glucoside	4.10E+00	Up	2.03
*flavone*			
luteolin	2.17E−09	Down	−28.78
baicalein	6.73E−02	Down	−3.89
mosloflavone	8.14E+00	Up	3.02
chrysin	8.54E+00	Up	3.09
*isoflavone*			
glycitein	6.61E+00	Up	2.72
6″-*O*-malonylgenistin	4.35E+00	Up	2.12
daidzein	1.24E+01	Up	3.63
biochanin A	8.31E+00	Up	3.06
formononetin	2.60E+01	Up	4.70
genistein	1.49E−01	Down	−2.74
afrormosin	1.37E+01	Up	3.78
*organic compound*			
benzeneacetaldehyde	2.51E+00	Up	1.33
methyl salicylate	2.12E+08	Up	27.66
*oligosaccharide*			
quercetin-3-*O*-glucosyl-rutinoside	1.67E+07	Up	23.99
*phenolic glycoside*			
methyl salicylate-*O*-[rhamnosyl-(1 → 6)-glucoside]	8.09E+00	Up	3.02
astragalin	3.38E−01	Down	−1.57
isorhamnetin glycoside	5.08E+07	Up	25.59
isorhamnetin glycoside	3.36E+09	Up	31.65
isorhamnetin glycoside	2.73E+09	Up	31.35
isorhamnetin glycoside	9.03E+07	Up	26.43
kaempferol-3-*O*-*β*-D-glucopyranosyl(1 → 2)-*O*-[*α*-L-rhamnopyranosyl(1 → 6)]-*β*-D-galactopyranoside	1.81E−02	Down	−5.79
kaempferol-3-*O*-*β*-D-glucopyranosyl(1 → 2)-*O*-[*α*-L-rhamnopyranosyl(1 → 6)]-*β*-D-glucopyranoside	1.47E−02	Down	−6.09
kaempferol-3-*O*-digalactopyranoside	4.66E−02	Down	−4.42
kaempferol-3-*O*-diglucopyranoside	3.44E−02	Down	−4.86
kaempferol-3-*O*-*β*-D-(2,6-di-*O*-*α*-L-rhamnopyranosyl)-galactopyranoside	1.26E−02	Down	−6.31

We observed a significant variation in the production of methyl salicylate (Table 4), which was superior to the resistant cultivar (PI 227682). This volatile molecule can interfere with herbivore behavior, decrease feeding performance, or act as an indirect resistance by attracting natural herbivore enemies, as demonstrated by Mallinger et al. (2011) in the interactions between *G. max* plants with soybean aphids, syrphid flies, and green lacewings.

**Figure 4:**
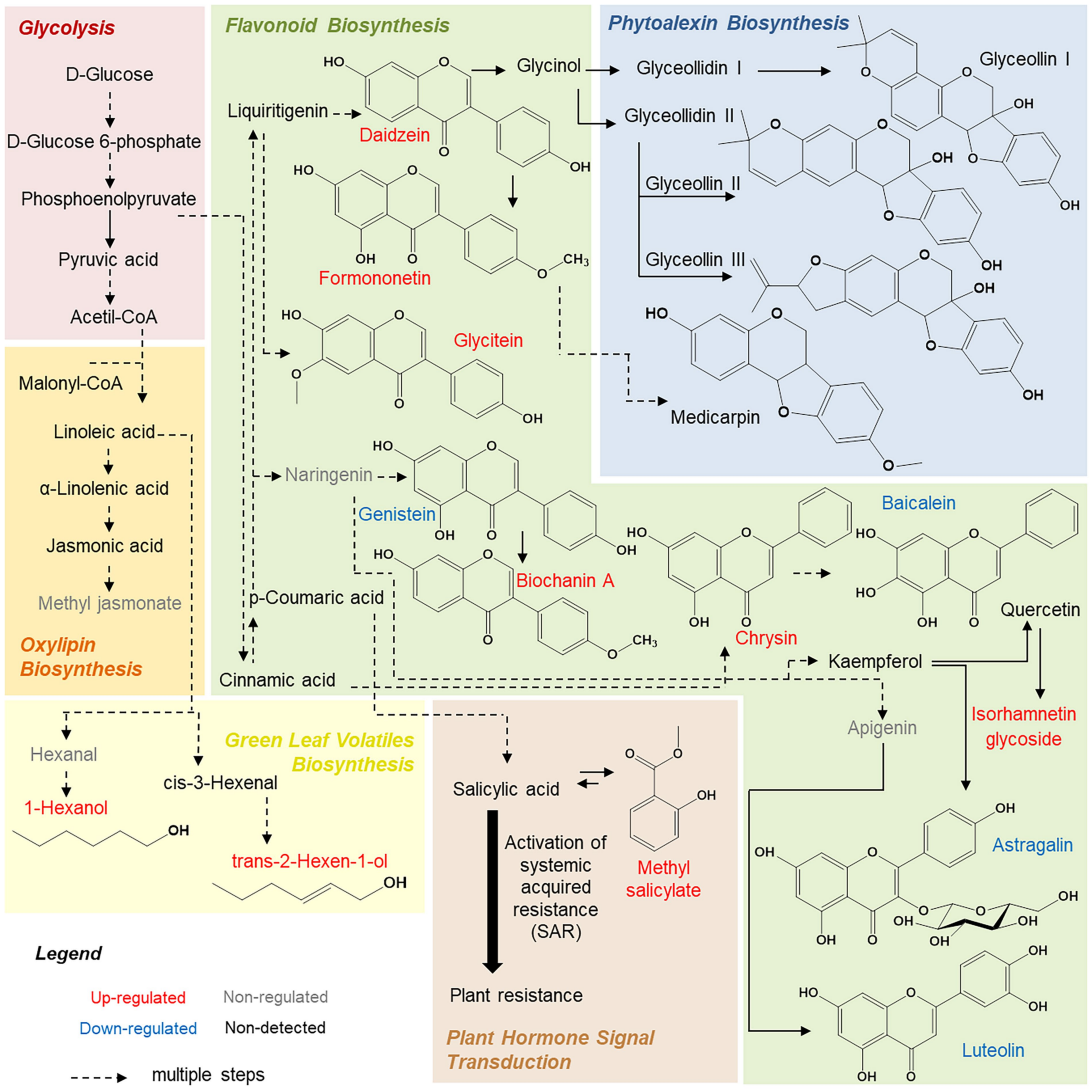
Summary of metabolic pathways predicted from changes in *Glycine max* leaf chemical profile.

Dempsey and Klessig (2012) and Derksen et al. (2013) also described methyl salicylate as a central molecule in systemic acquired resistance mechanisms. This molecule is a methyl ester of salicylic acid, which is biologically inactive in *G. max* leaves (Figure 4). When the plant is under environmental stress, methyl salicylate is transported to the leaves (systemic tissue) by floem, where it can be converted through a series of signaling reactions into salicylic acid and begin systemic defense mechanisms (Dempsey and Klessig, 2012; Derksen et al., 2013).

In this study, *G. max* plants were not stressed by pathogens or herbivores. Thus, a significant accumulation of methyl salicylate in its inactive form may be a resistance strategy in plants since higher quantities may allow a quicker defense response in a stressful environment when compared to susceptible cultivars (Pastor et al., 2013).

The compounds octanal, nonanal, heptadecanal, indole, 1-hexanol, *trans*-3-hexen-1-ol, and *trans*-2-hexen-1-ol showed a higher expression in the susceptible cultivar when compared to the resistant one (Table 4). Intact plants generally emit these compounds only in trace quantities. Nevertheless, they can be produced in larger quantities when plants are artificially injured or attacked by herbivores (Tanaka et al., 2018).

Some volatile molecule action mechanisms are not yet fully understood. There are, however, pieces of evidence that volatile compounds in green leaves, such as 1-hexanol, *trans*-2-hexen-1-ol, or methyl salicylate, are released as a botanical indirect defense mechanism through the attraction of natural predators of herbivores which act directly upon insect pests (Scala et al., 2013; Tanaka et al., 2018). These compounds can also help strengthen plant cell walls by increasing the lignin content in leaves. Moreover, they can help accumulate phytoalexins and act as intermediates in the methyl jasmonate pathway, an essential phytohormone in regulating plant responses to biotic and abiotic stresses (Zeringue, 1992; Kishimoto et al., 2006; Christensen et al., 2013).

We also highlight some molecules from isoflavone biosynthesis, such as biochanin A, daidzein, and formononetin (Table 4). These compounds are connected to the botanical defense mechanism and accumulate in plants during biotic or abiotic stresses (Křížová et al., 2019). For example, these compounds, when released, can induce genes from plant nodulation or be metabolized to produce coumestrol, medicarpin, glyceollins, or glycosylated flavonoids (Křížová et al., 2019), as illustrated in Figure 4.

Although the final products of these metabolic pathways did not present significant variations in our study, the higher proportion of their precursors in the resistant cultivar indicates a greater accumulation capacity of these intermediate substances during development than susceptible ones.

Finally, we highlight that the resistant cultivar presented a higher accumulation of glycosylated isorhamnetin molecules (Table 4; Figure 4). This information is interesting because we previously demonstrated that susceptible cultivars showed a higher quantity of glycosylated kaempferol derivatives. Lattanzio et al. (2000) observed a similar relationship between resistance and susceptibility against aphids, and the content of flavonoid glycosides in Vigna leaves indicated higher bioactivity of isorhamnetin (a molecule derived from the quercetin, Figure 4) when compared to kaempferol. Thus, reversible glycosylation of isorhamnetin molecules could also be a *G. max* resistance mechanism, facilitating transport and storage of the molecules. Additionally, the toxicity of these molecules to the plant is reduced (Le Roy et al., 2016).

## Conclusion

We have provide evidence that the biosynthesis of lipids and isoflavones in *G. max* may directly relate to the constitutive resistance presented by plants against *S. cosmioides* caterpillars. Data on volatile and nonvolatile compounds from multiplatform analysis suggest that the main soybean defense strategy may involve a higher cell membrane lipid biosynthesis, storage of molecules in inactive form, and several fatty acid products that serve as defense signals, including green leaf volatiles. Therefore, resistant plants would be in a state of alert, which prepares them for faster and more robust activation of defense in response to herbivory. These results increase our knowledge about the interaction between *G. max* plants and herbivore insects such as *S. cosmioides* and encourage the applications of constitutive defense metabolites for the biorational control of pest insects. Finally, the study of the mechanism behind *G. max* plant resistance was possible after the standardization of extraction methodologies that improved sample yield with more effective extraction for most metabolites.

## Data availability statement

The original contributions presented in the study are included in the article/Supplementary material, further inquiries can be directed to the corresponding author.

## Author contributions

MA designed the experiments, acquired, analyzed, interpreted the data, and drafted the manuscript. MF, CF, and AB collaborated on the biological assays. RC supported the chemometric analyses and interpretations. MS and JF participated in the data discussion and financial support. MF conceived the study, analyzed and interpreted the data, and critically read and revised the manuscript. All authors contributed to the article and approved the submitted version.

## Funding

The authors are grateful for the financial support received from the National Council for Scientific and Technological Development – CNPq (grant numbers 406537/2021–6, 429404/2018–2, 307800/2021–0, 465357/2014–8); the São Paulo Research Foundation – FAPESP (grant numbers 2018/00340–0, 2018/21201–8, 2014/50918–7, 2021/11878–3); and Coordination for the Improvement of Higher-Level Personnel (CAPES; Finance Code 001).

## Conflict of interest

The authors declare that the research was conducted in the absence of any commercial or financial relationships that could be construed as a potential conflict of interest.

## Publisher’s note

All claims expressed in this article are solely those of the authors and do not necessarily represent those of their affiliated organizations, or those of the publisher, the editors and the reviewers. Any product that may be evaluated in this article, or claim that may be made by its manufacturer, is not guaranteed or endorsed by the publisher.
