# Supplementary material for: Exploring chemical diversity in Glycine max cultivars: A multivariate approach in the search for bioactive compounds against Spodoptera cosmioides
Source: Front Plant Sci. 2022 Sep 2;13:987782. doi: 10.3389/fpls.2022.987782 (PMC9478465; doi:10.3389/fpls.2022.987782)

Supplementary Material

Exploring chemical diversity in *Glycine max* cultivars: a multivariate approach in the search for bioactive compounds against *Spodoptera cosmioides*

Maria Clara Santana Aguiar[1], Marcelo Mueller de Freitas[2], Carlos Alessandro de Freitas[2], Arlindo Leal Boiça Júnior[2], Renato Lajarim Carneiro[3], Maria Fátima das Graças Fernandes da Silva[1], João Batista Fernandes[1], Moacir Rossi Forim[1*]

[1]Laboratory of Natural Products, Department of Chemistry, Universidade Federal de São Carlos, São Carlos, Brazil

[2]Laboratory of Plant Resistance to Insects, Department of Agricultural Sciences, Universidade do Estado de São Paulo, Jaboticabal, Brazil

[3]Laboratory of Applied Chemometrics, Department of Chemistry, Universidade Federal de São Carlos, São Carlos, Brazil

*** Correspondence:**
Corresponding Author
mrforim@ufscar.br

Supplementary Table 1. Matrix representation of the factorial design and the response obtained for solid-liquid extraction.

Test nº	Codel levels				Response variables				Dg
	A	B	C	D	Number of compounds	Σ(peak area/internal standard area) (1-5 min)	(5-10 min)	(10-15 min)	
1	1	1	1	1	52	11.4	44.1	15.5	0.93
2	-1	1	1	1	41	10.4	37.4	10.2	0.45
3	1	-1	1	1	41	11.4	37.6	10.3	0.52
4	-1	-1	1	1	41	11.8	37.4	10.7	0.56
5	1	1	-1	1	39	12.3	43.5	10.0	0.64
6	-1	1	-1	1	40	9.8	32.1	7.5	0.24
7	1	-1	-1	1	45	10.6	34.8	8.6	0.44
8	-1	-1	-1	1	48	10.1	37.8	10.1	0.54
9	1	1	1	-1	39	11.5	43.1	11.2	0.61
10	-1	1	1	-1	37	10.3	29.8	8.1	0.20
11	1	-1	1	-1	43	11.4	31.9	8.4	0.41
12	-1	-1	1	-1	35	11.3	29.0	7.2	0.21
13	1	1	-1	-1	41	12.1	41.5	10.8	0.65
14	-1	1	-1	-1	44	11.4	29.3	8.0	0.37
15	1	-1	-1	-1	45	12.5	30.0	8.9	0.49
16	-1	-1	-1	-1	43	10.8	28.7	6.2	0.26
17	0	0	0	0	49	10.5	29.3	10.5	0.45
18	0	0	0	0	48	10.7	37.6	9.1	0.55
19	0	0	0	0	48	8.7	41.1	10.7	0.51

A: temperature (25, 50 e 70ºC); B: extractor mixture - methanol solution (50:50, 30:70 and 10:90% v v^{-1}); C: time (5, 20 e 35 min); D: number of extractions (1, 2 and 3); -1: lower level; + 1: higher level; Dg: Global desirability.

Supplementary Table 2. Matrix representation of the factorial design and the response obtained for solid-liquid extraction using ultrasound bath.

Test nº	Codel levels A	B	C	Number of compounds	Response variables: Σ(peak area/internal standard area) (1-5 min)	(5-10 min)	(10-15 min)	Dg
1	1	1	1	39	7.29	29.6	7.71	0.53
2	-1	1	1	39	10.1	39.1	10.7	0.83
3	1	-1	1	45	10.1	25.9	9.04	0.71
4	-1	-1	1	44	12.4	40.9	7.18	0.89
5	1	1	-1	31	7.62	24.4	6.07	0.37
6	-1	1	-1	19	6.49	14.2	2.29	0.00
7	1	-1	-1	41	10.2	28.2	5.88	0.61
8	-1	-1	-1	39	10.1	28.7	5.15	0.57
9	0	0	0	39	9.70	31.07	5.39	0.58
10	0	0	0	38	9.53	30.23	5.22	0.55
11	0	0	0	39	9.27	32.53	5.53	0.58

A: time (5, 20 and 35 min); B: extractor mixture - methanol solution (50:50, 30:70 and 10:90% v v^{-1}); C: number of extractions (1, 2 and 3); -1: lower level; + 1: higher level; Dg: Global desirability.

Supplementary Figure 1. Total ion chromatograms of nonvolatile compounds from *Glycine max* leaves using the optimized conditions of solid-liquid extraction and solid-liquid extraction assisted by an ultrasound bath.

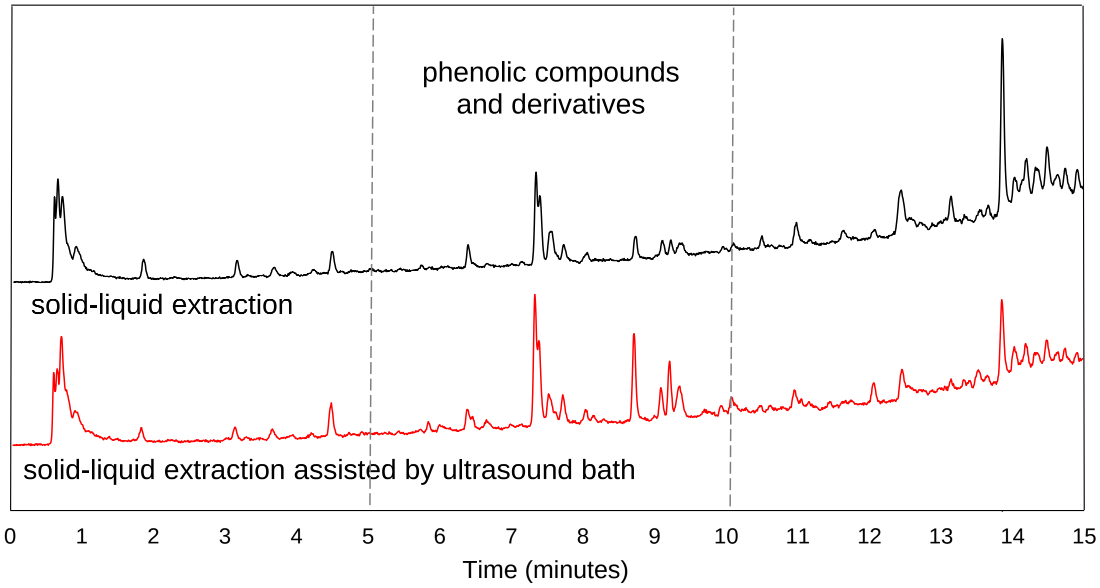

Supplementary Figure 2. Contour curves for solid-liquid extraction (a) and solid-liquid extraction assisted by ultrasound bath (b).

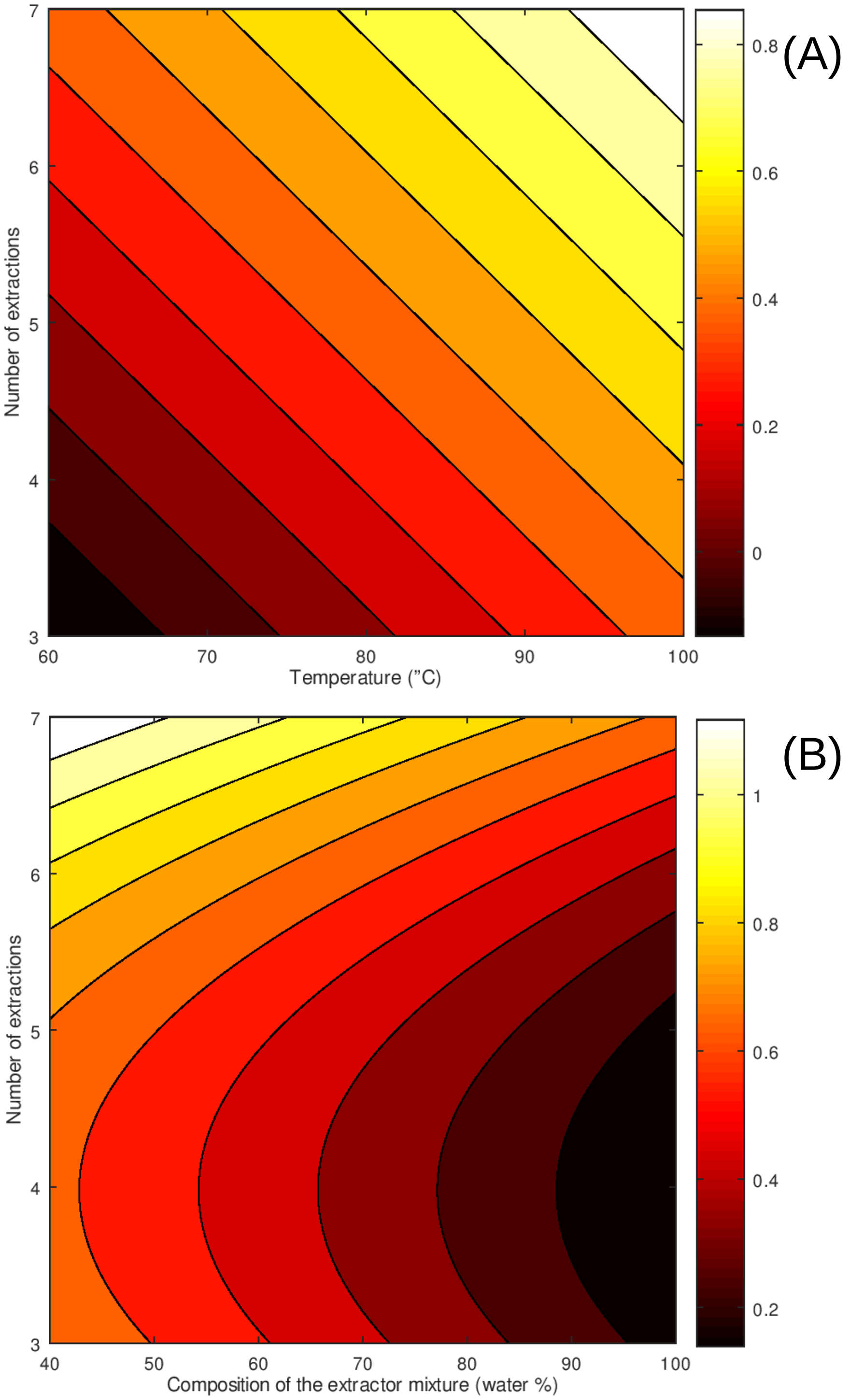

Supplementary Table 3. Matrix representation of the factorial planning and the response obtained for static headspace extraction.

Test nº	Codel levels			Response variables				Dg
	A	B	C	Number of compounds	Σ(Relative abundance) (1-7 min)	(7-15 min)	(15-30 min)	
1	-1	-1	-1	28	75.0	12.3	12.8	0.34
2	1	-1	-1	20	80.7	16.7	2.6	0.28
3	-1	1	-1	52	58.2	37.3	4.5	0.56
4	1	1	-1	48	82.3	22.7	5.0	0.54
5	-1	-1	1	18	84.1	19.1	18.7	0.39
6	1	-1	1	20	71.5	22.6	5.9	0.30
7	-1	1	1	40	67.3	40.4	6.3	0.55
8	1	1	1	53	76.8	48.6	8.6	0.75
9	0	0	0	31	56.0	27.6	9.4	0.36
10	0	0	0	31	62.1	28.9	9.0	0.39
11	0	0	0	32	62.1	31.7	7.2	0.41

A: time (5, 15 e 25); B: temperature (40, 80 e 120ºC); C: saturation with glycerol (0, 0.5 e 1.0 g); -1: lower level; + 1: higher level; Dg: Global desirability.

Supplementary Figure 3. Total ion chromatogram of volatile compounds from *Glycine max* leaves using the optimized conditions of static headspace extraction.

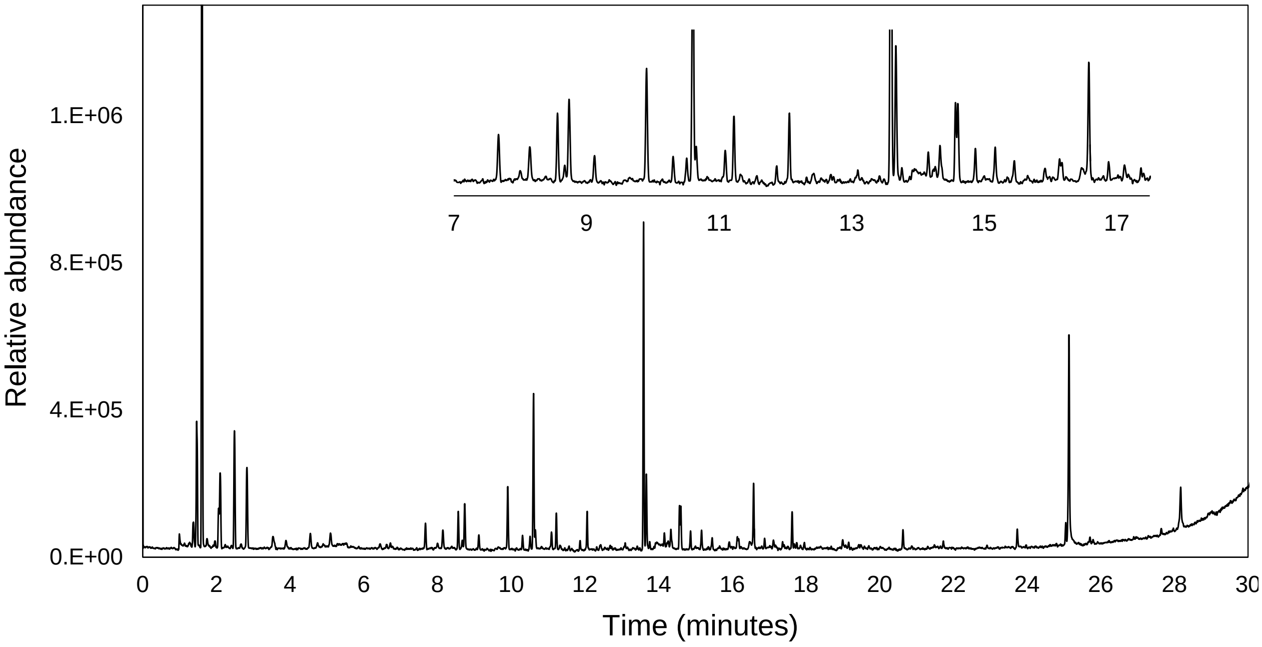

Supplementary Figure 4. Comparison of the total ion chromatograms of nonvolatile compounds from leaves of *Glycine max* of ten cultivars using the optimized conditions of solid-liquid extraction assisted by ultrasound bath.

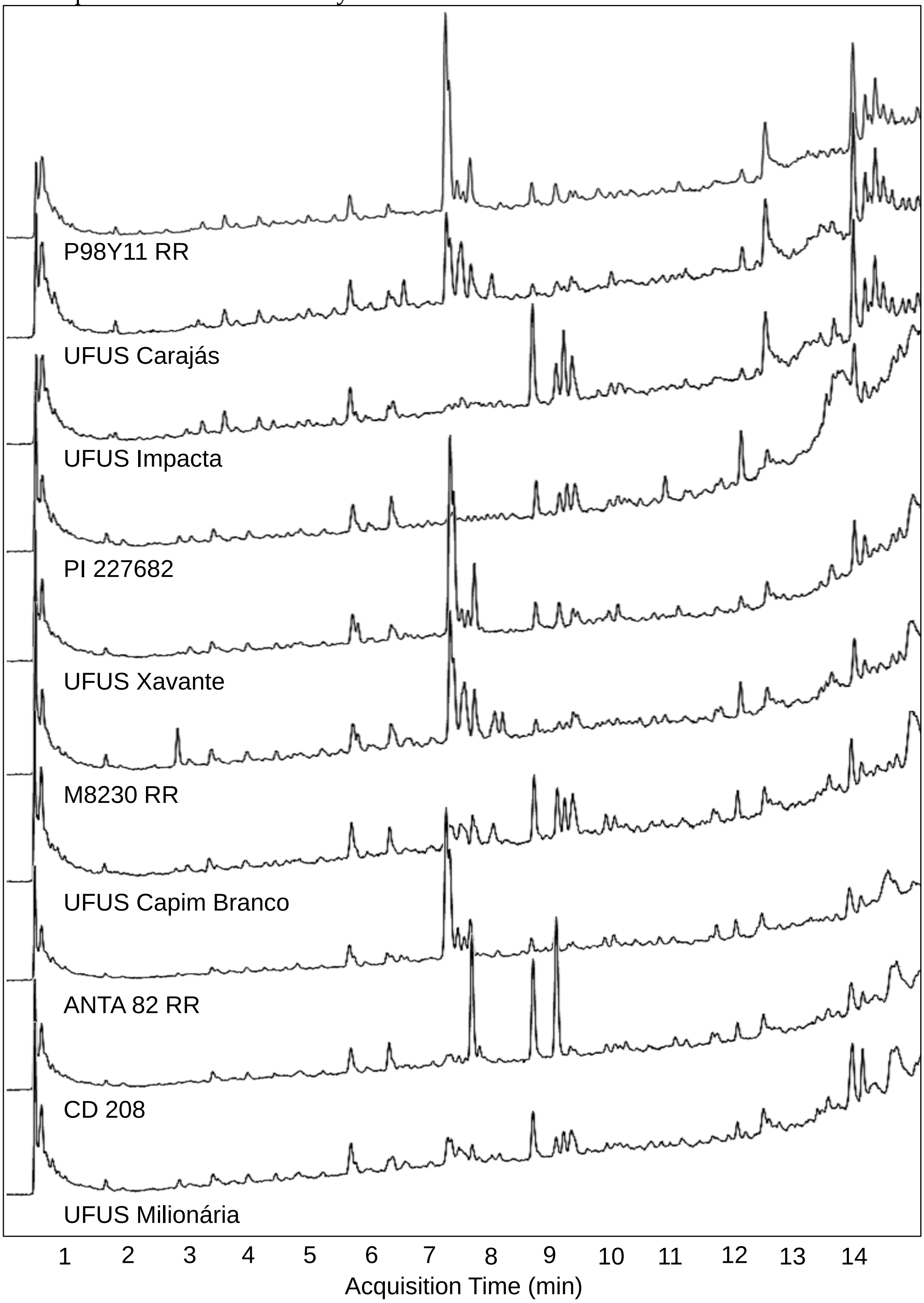

Supplementary Figure 5. Comparison of the total ion chromatograms of volatile compounds from leaves of *G. max* of ten cultivars using the optimized conditions of static headspace extraction.

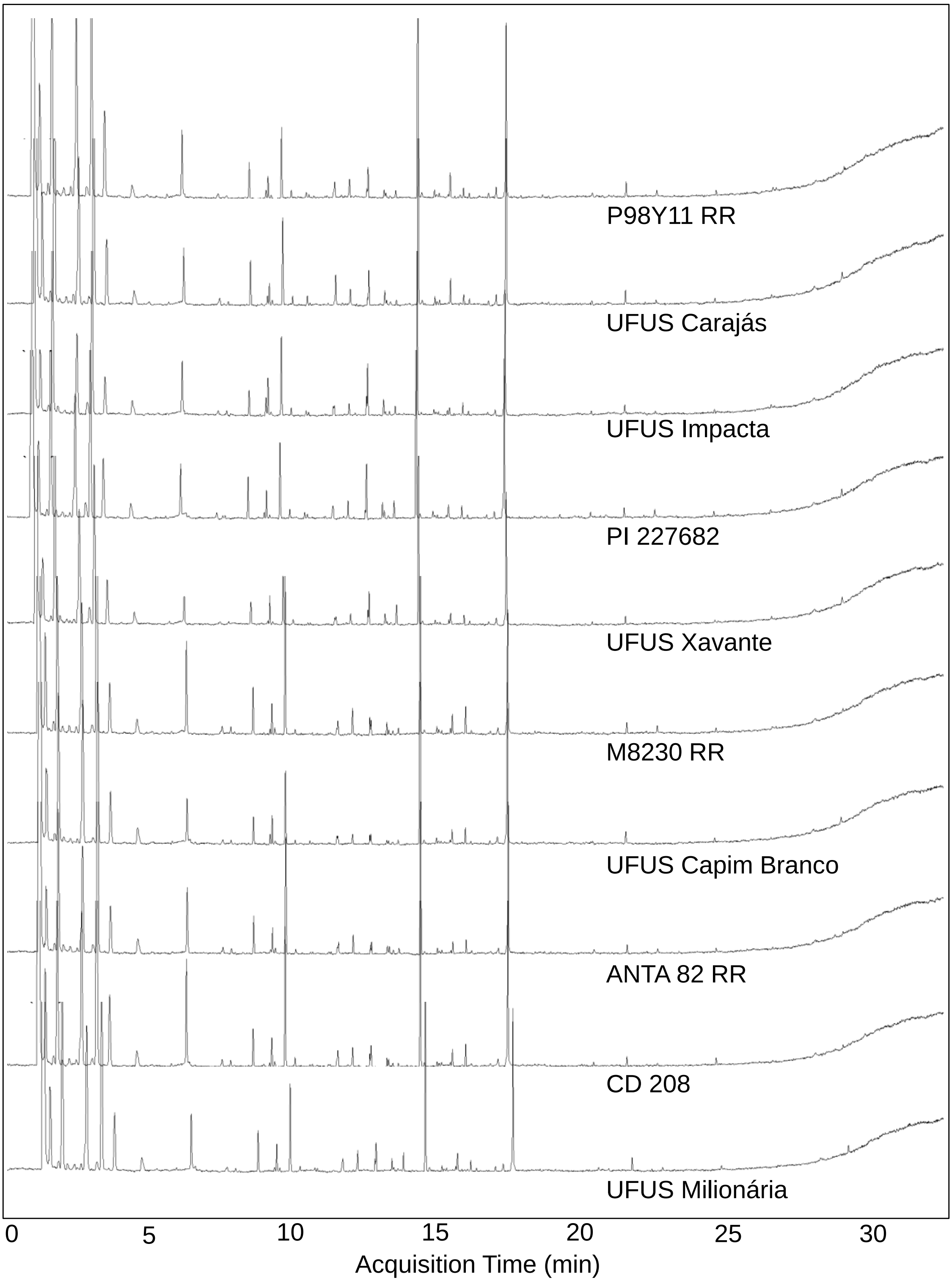

Supplementary Table 4. Nonvolatile compounds were detected in *Glycine max* leaves extract (Cavaliere et al., 2007; Ho et al., 2002; Jeon et al., 2012; Klejdus et al., 2005; Salerno et al., 2017; Song et al., 2014; Zanzarin et al., 2019).

Compound identification		Main fragments	Detected precursor ions			Molecular formula	Error (ppm)
			$(M+H)^+$	$(M+Na)^+$	$(M+NH_4)^+$		
Caffeine (Internal Standard)	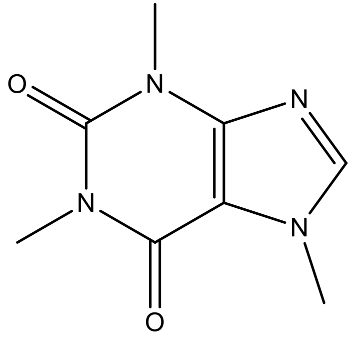	138, 123, 110	195.0873	217.0645	-	$C_8H_{10}N_4O_2$	1.41
Indole	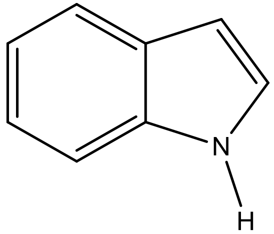	117	118.0646	140.0497	-	C_8H_7N	3.29
Trigonelline	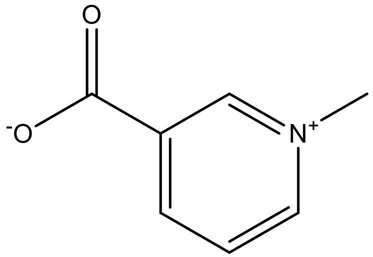	136, 123, 110	138.0548	-	-	$C_7H_7NO_2$	0.57

Tuberonic acid glucoside	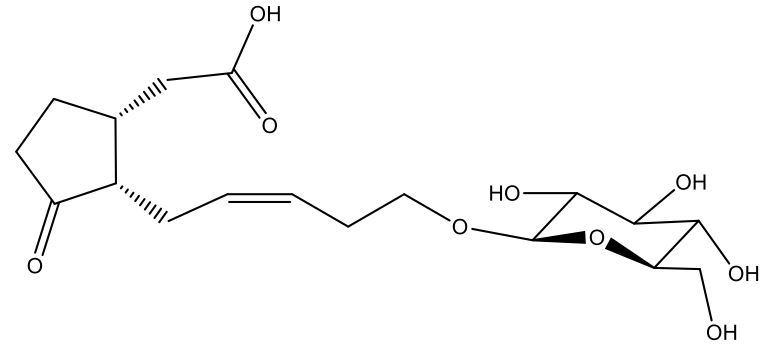	367, 209	-	411.1619	-	$C_{18}H_{28}O_9$	2.00
Tetradecanoic acid	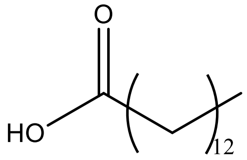	229, 209	-	-	246.2427	$C_{14}H_{28}O_2$	-0.11
16-Hydroxy hexadecanoic acid	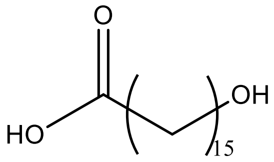	274, 258	-	-	290.2686	$C_{16}H_{32}O_3$	1.26
Palmitic amide	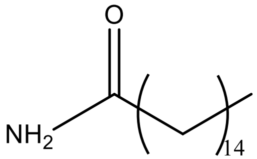	212	256.2634	-	-	$C_{16}H_{33}NO$	0.21

Luteolin	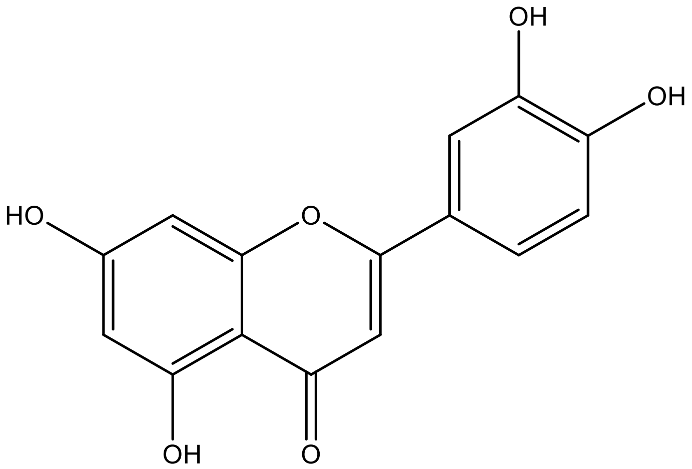	133, 128, 107	287.0547	-	-	$C_{15}H_{10}O_6$	0.88
3-*O*-methylquercetin	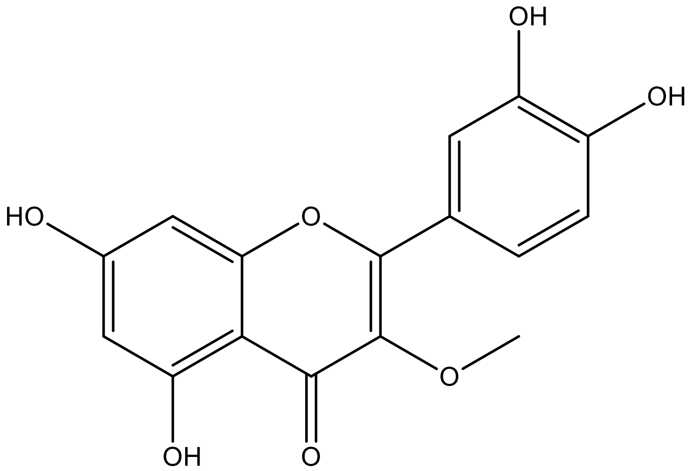	302, 274, 229	317.0646	-	-	$C_{15}H_{10}O_7$	3.12

Baicalein	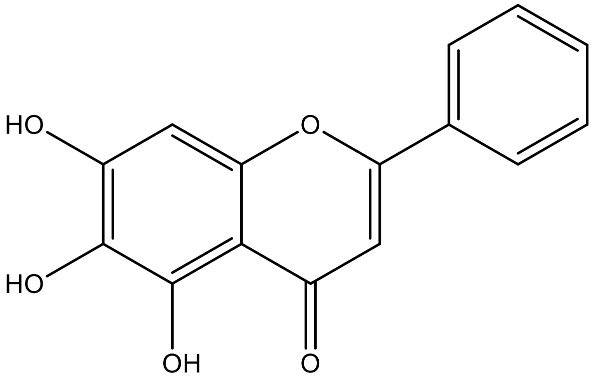	141, 123, 115, 105	271.0597	-	-	$C_{15}H_{10}O_5$	1.11
Apigenin	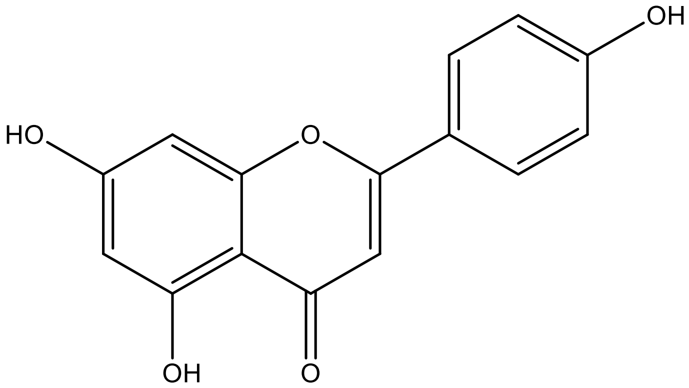	197, 187, 153, 131, 115, 109	271.0598	-	-	$C_{15}H_{10}O_5$	0.99
Mosloflavone	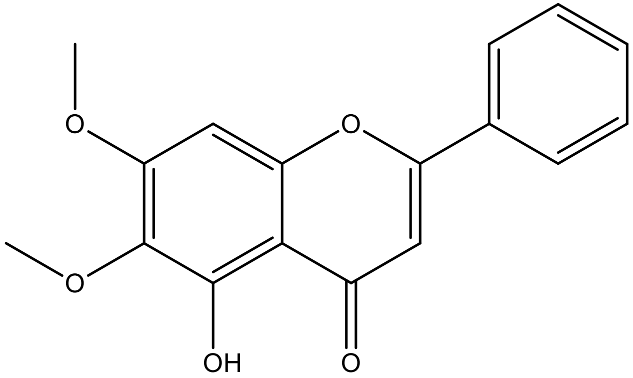	284	299.0913	321.0727	-	$C_{17}H_{14}O_5$	0.64

Naringenin	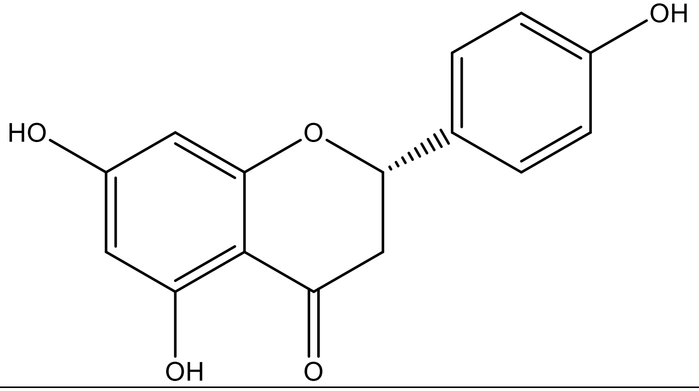	153, 147	273.0752	-	-	$C_{15}H_{12}O_5$	1.51
Chrysin	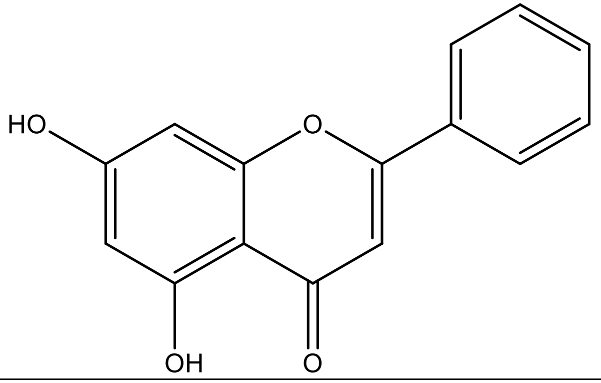	115, 103	255.0651	-	-	$C_{15}H_{10}O_4$	0.3
Glycitein	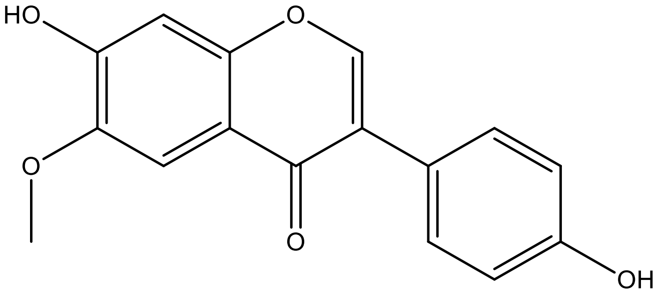	270, 242, 128, 115, 107	285.0754	-	-	$C_{16}H_{12}O_5$	0.84

6"-*O*-Malonylgenistin	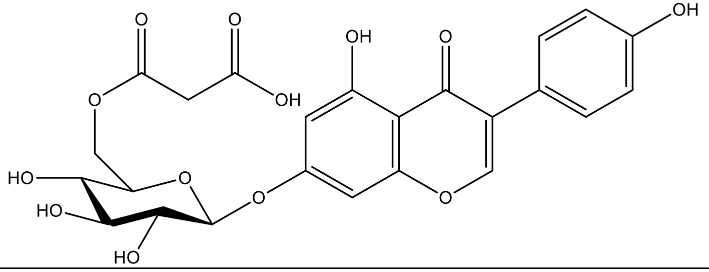	271	518.1053	541.09410	-	$C_{24}H_{22}O_{13}$	1.75
Daidzein	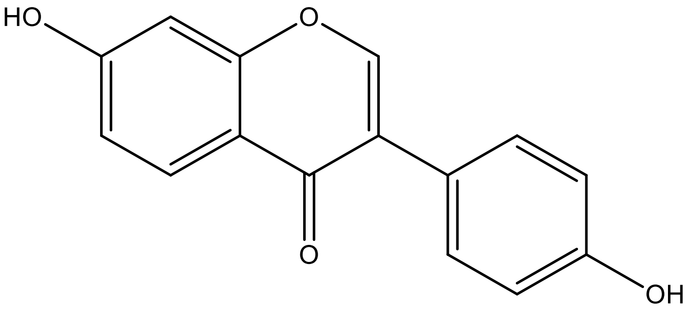	227, 199, 152, 128, 115, 107, 103	255.0655	-	-	$C_{15}H_{10}O_4$	-0.27
Biochanin A	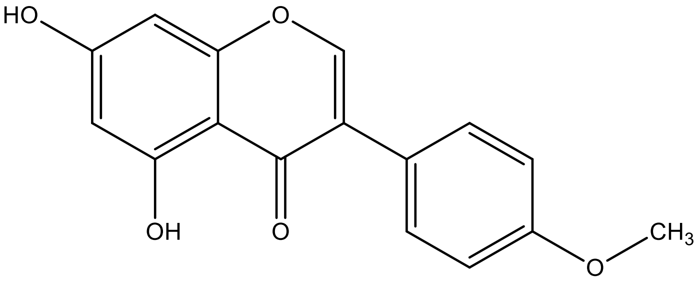	270, 253, 213	285.0751	-	-	$C_{16}H_{12}O_5$	1.96
Formononetin	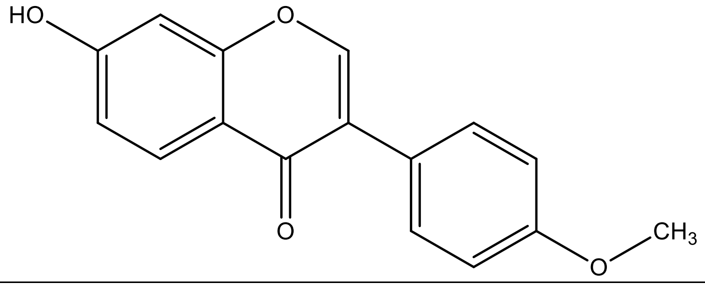	253, 226, 213	-	269.0805	-	$C_{16}H_{12}O_4$	1.57

Genistein	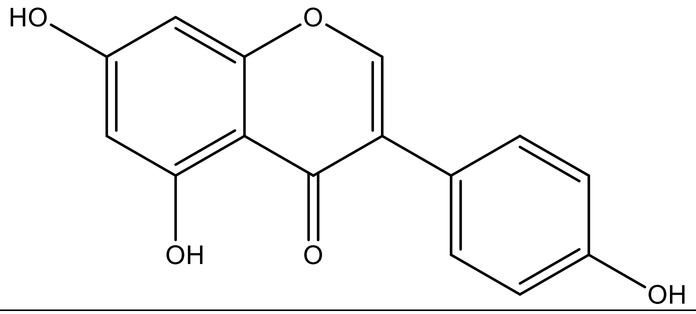	243, 159, 152, 115, 107	271.0600	-	-	$C_{15}H_{10}O_5$	0.49
Afrormosin	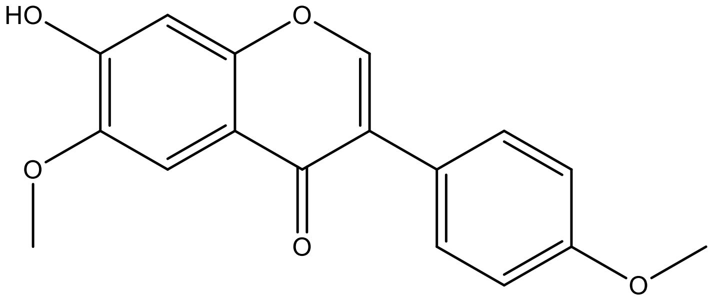	284, 141, 128, 121, 117	299.0912	321.0731	-	$C_{17}H_{14}O_5$	-0.17
Methyl jasmonate	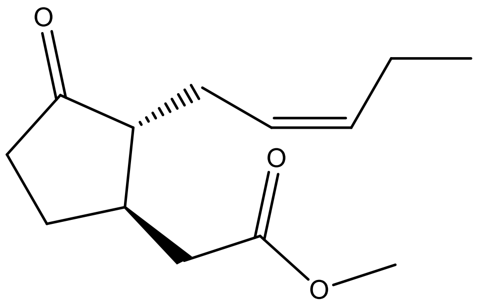	147, 133, 109	225.1483	-	-	$C_{13}H_{20}O_3$	2.13

Quercetin-3-*O*-glucosyl-rutinoside	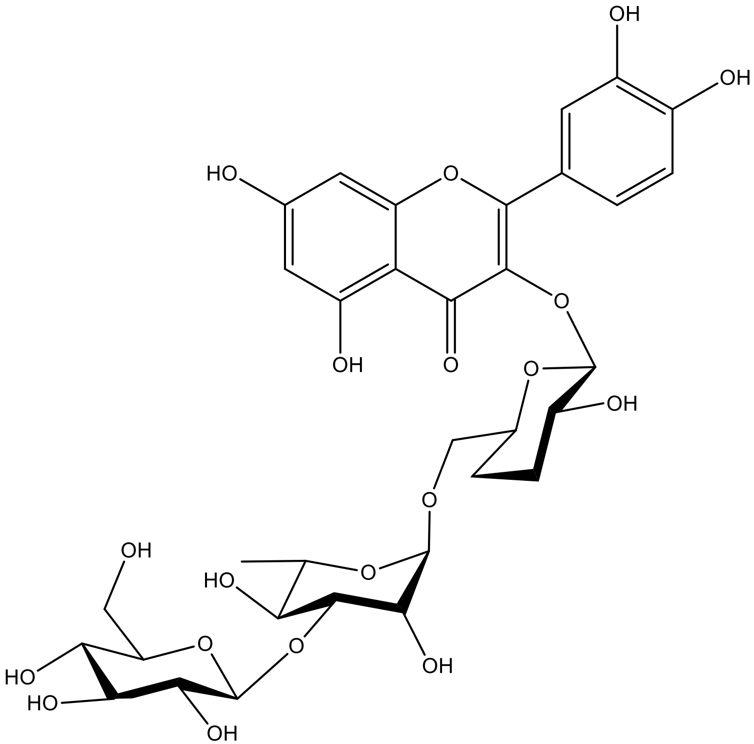	465, 347, 303, 279, 153, 117, 107	-	795.1948	-	$C_{33}H_{40}O_{21}$	0.86

Methyl salicylate-*O*-[rhamnosyl-(1→6)-glucoside]	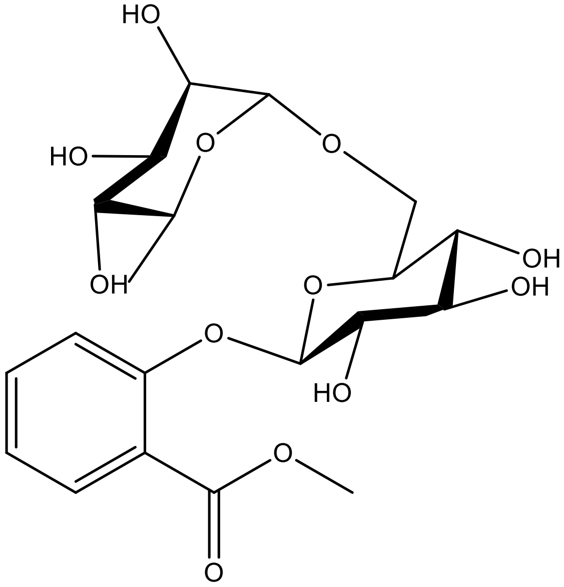	-	-	483.147	-	$C_{20}H_{28}O_{12}$	0.69
Kaempferol-3-*O*-β-D-glucopyranosyl(1→2)-*O*-[α-L-rhamnopyranosyl(1→6)]-β-D-galactopyranoside	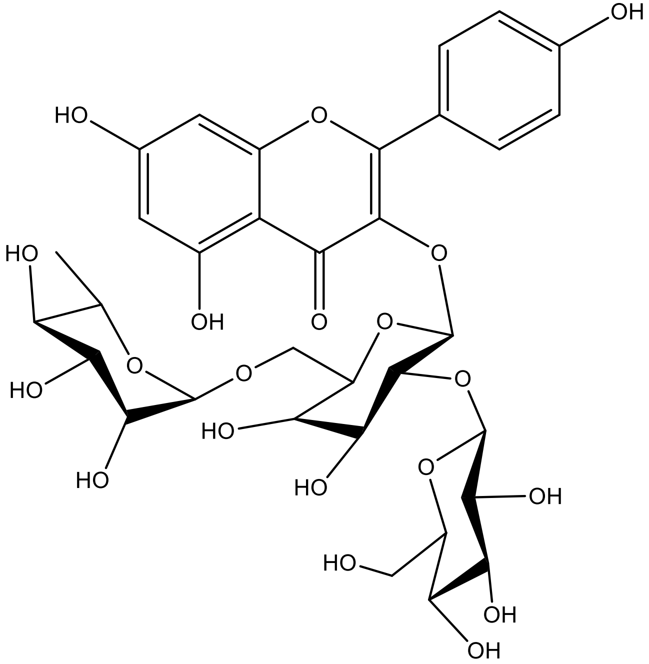	495, 449, 287	756.2107	779.2000	-	$C_{33}H_{40}O_{20}$	0.91

Kaempferol-3-*O*-β-D-glucopyranosyl(1→2)-*O*-[α-L-rhamnopyranosyl(1→6)]-β-D-glucopyranoside	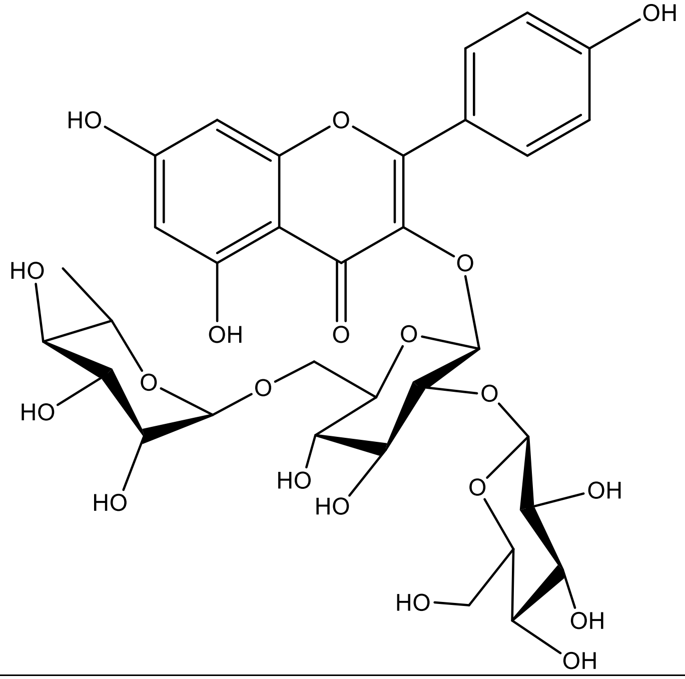	497, 449, 287	756.2103	779.1996	-	$C_{33}H_{40}O_{20}$	1.31

Kaempferol-3-*O*-digalactopyranoside	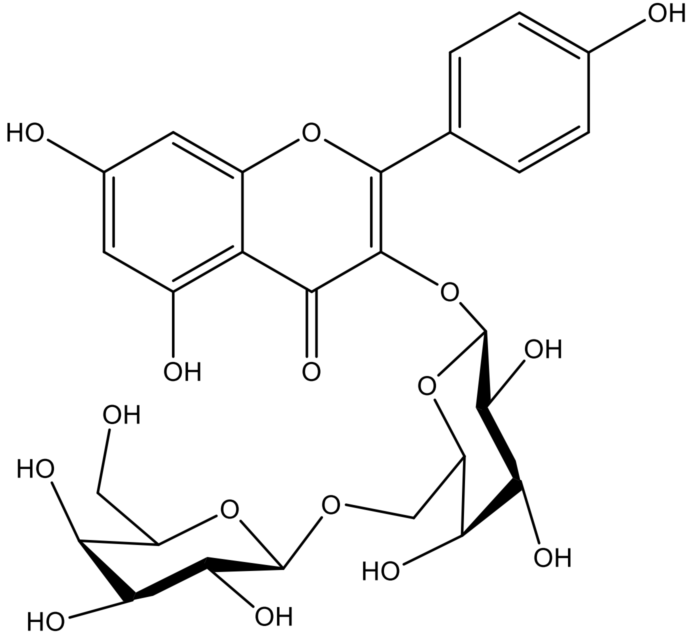	287	610.1522	633.1413	-	$C_{27}H_{30}O_{16}$	2.51
Kaempferol-3-*O*-diglucopyranoside	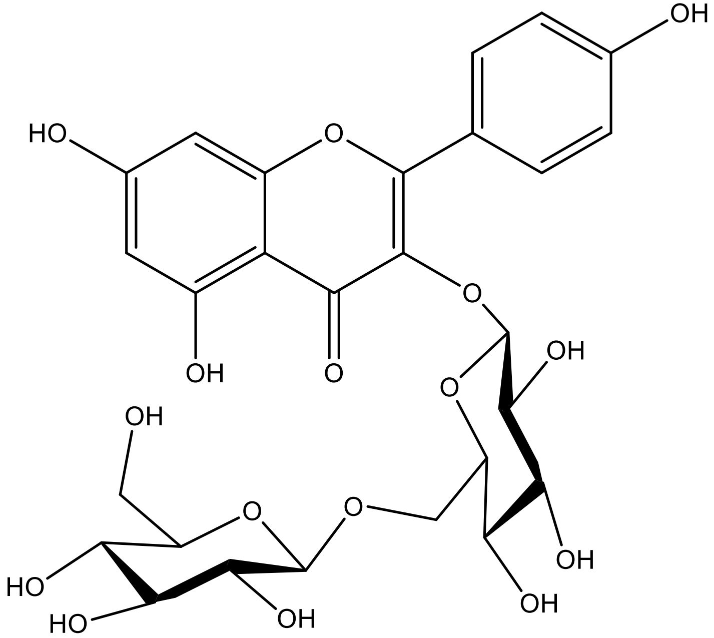	287	610.1525	633.1422	-	$C_{27}H_{30}O_{16}$	1.32

Kaempferol-3-*O*-β-D-(2,6-di-*O*-α-L-rhamnopyranosyl) galactopyronoside	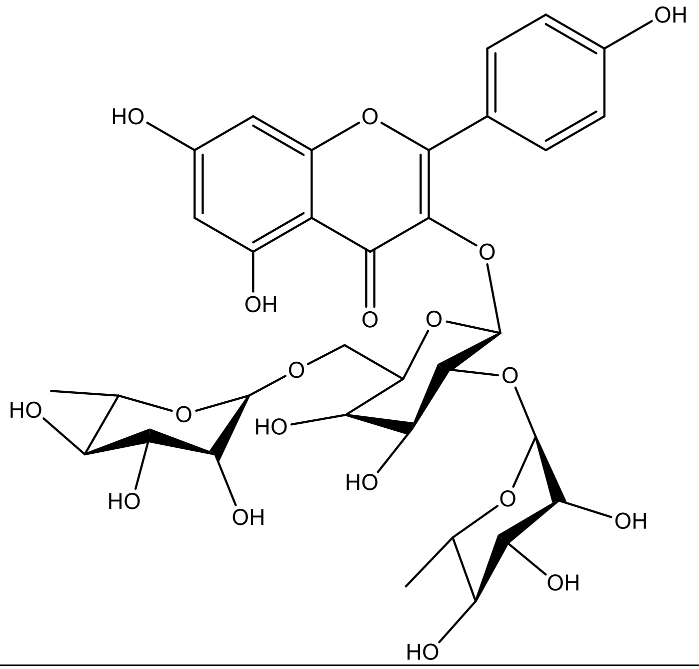	287	740.2150	763.2040	-	$C_{33}H_{40}O_{19}$	2.06

Kaempferol-3-*O*-α-L-rhamnopyranosyl (1→6)-β-D-galactopyronoside	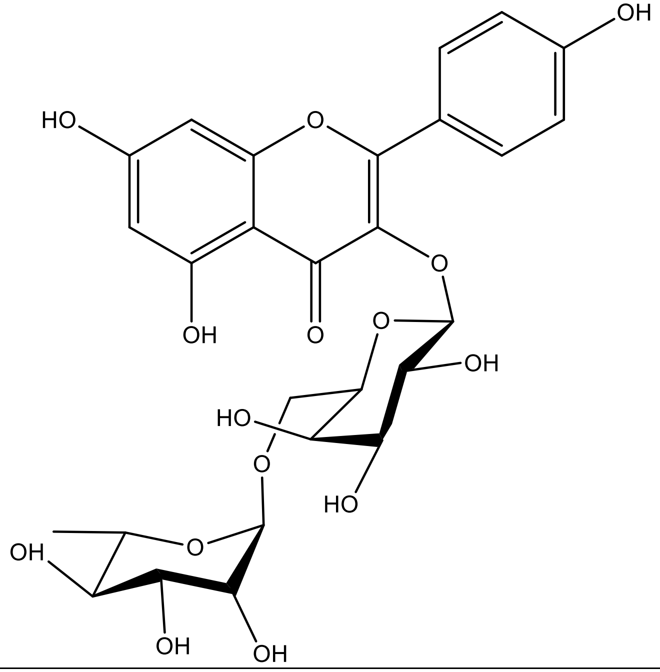	287	594.1582	617.1475	-	$C_{27}H_{30}O_{15}$	0.56
Astragalin	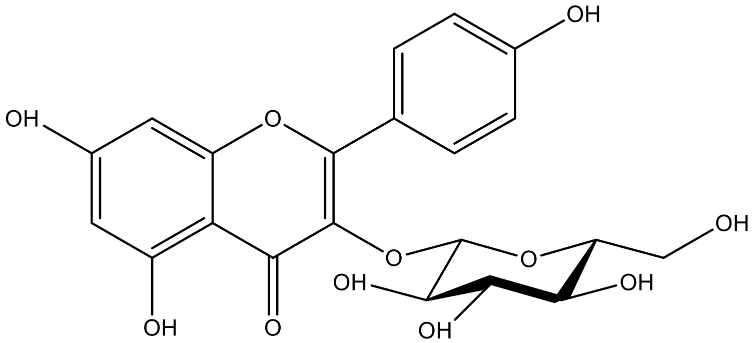	287, 165, 153, 121	-	471.0884	-	$C_{21}H_{20}O_{11}$	3.29

Kaempferol-3-*O*-α-L-rhamnopyranosyl(1→6)-β-D-glucopyronoside	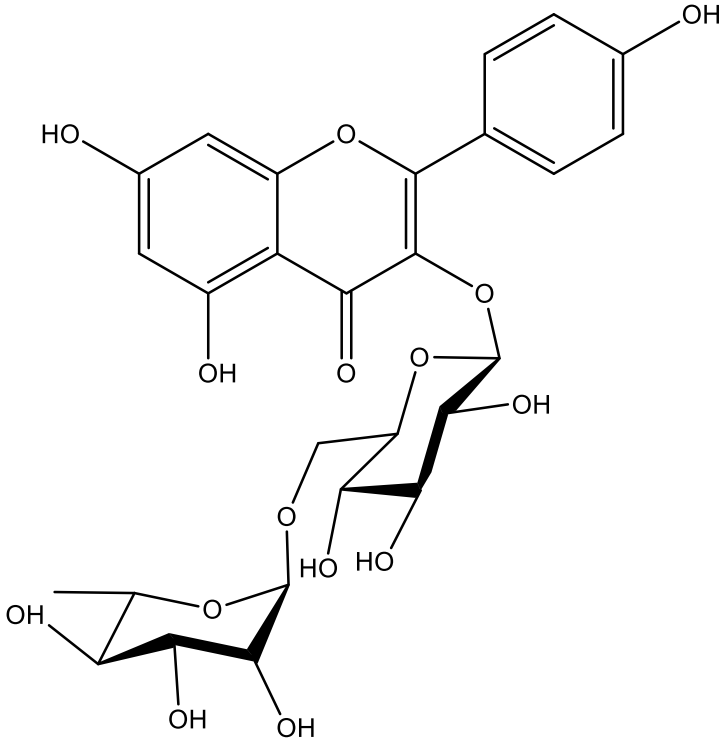	287	594.1577	617.1472	-	$C_{27}H_{30}O_{15}$	1.16
Kaempferol glycoside		287	-	471.0884	-	$C_{21}H_{20}O_{11}$	3.42
Isorhamnetin glycoside		317	-	501.0984	-	$C_{22}H_{22}O_{12}$	3.76
Isorhamnetin glycoside		317	-	501.0989	-	$C_{22}H_{22}O_{12}$	3.37
Isorhamnetin glycoside		317	-	617.1463	-	$C_{27}H_{30}O_{15}$	3.52
Isorhamnetin glycoside		317	-	647.1564	-	$C_{28}H_{32}O_{16}$	2.8
Isorhamnetin glycoside		317	-	647.1580	-	$C_{28}H_{32}O_{16}$	0.27
Isorhamnetin glycoside		317	-	647.1592	-	$C_{28}H_{32}O_{16}$	-1.86

Hexadecasphinganine	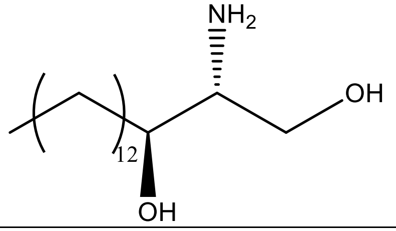	256	274.2739	-	-	$C_{16}H_{35}NO_2$	0.65
Phytosphingosine	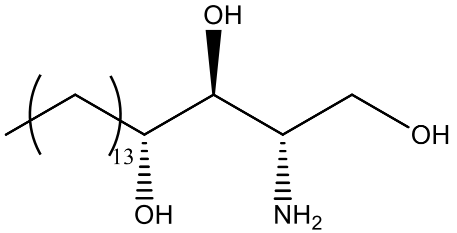	300	318.3006	-	-	$C_{18}H_{39}NO_3$	-0.85
Sesquiterpene		-	235.1690	-	-	$C_{15}H_{22}O_2$	1.46

Cavaliere, C., Cucci, F., Foglia, P., Guarino, C., Samperi, R., Laganà, A., 2007. Flavonoid profile in soybeans by high-performance liquid chromatography/tandem mass spectrometry. Rapid Commun. Mass Spectrom. 21, 2177–2187. https://doi.org/10.1002/rcm.3049

Ho, H.M., Chen, R.Y., Leung, L.K., Chan, F.L., Huang, Y., Chen, Z.Y., 2002. Difference in flavonoid and isoflavone profile between soybean and soy leaf. Biomed. Pharmacother. 56, 289–295. https://doi.org/10.1016/S0753-3322(02)00191-9

Jeon, H.Y., Seo, D.B., Shin, H.J., Lee, S.J., 2012. Effect of Aspergillus oryzae -challenged germination on soybean isoflavone content and antioxidant activity. J. Agric. Food Chem. 60, 2807–2814. https://doi.org/10.1021/jf204708n

Klejdus, B., Mikelová, R., Petrlová, J., Potěšil, D., Adam, V., Stiborová, M., Hodek, P., Vacek, J., Kizek, R., Kubáň, V., 2005. Evaluation of isoflavone aglycon and glycoside distribution in soy plants and soybeans by fast column high-performance liquid chromatography coupled with a diode-array detector. J. Agric. Food Chem. 53, 5848–5852. https://doi.org/10.1021/jf0502754

Salerno, G., Frati, F., Marino, G., Ederli, L., Pasqualini, S., Loreto, F., Colazza, S., Centritto, M., 2017. Effects of water stress on emission of volatile organic compounds by Vicia faba, and consequences for attraction of the egg parasitoid Trissolcus basalis. J. Pest Sci. (2004). 90, 635–647. https://doi.org/10.1007/s10340-016-0830-z

Song, H.H., Ryu, H.W., Lee, K.J., Jeong, I.Y., Kim, D.S., Oh, S.R., 2014. Metabolomics investigation of flavonoid synthesis in soybean leaves depending on the growth stage. Metabolomics 10, 833–841. https://doi.org/10.1007/s11306-014-0640-3

Zanzarin, D.M., Hernandes, C.P., Leme, L.M., Silva, E., Porto, C., Martin do Prado, R., Meyer, M.C., Favoreto, L., Nunes, E. de O., Pilau, E.J., 2019. Metabolomics of soybean green stem and foliar retention (GSFR) disease using mass spectrometry and molecular networking. Rapid Commun. Mass Spectrom. 1–8. https://doi.org/10.1002/rcm.8655

Supplementary Table 5. Volatile compounds detected in *Glycine max* leaves.

Compound identification		Molecular formula	Main fragments
Menthol (Internal Standard)	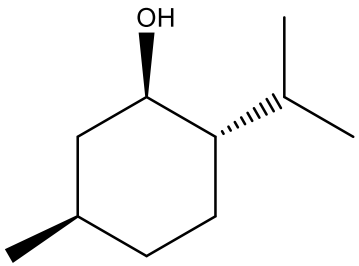	$C_{10}H_{20}O$	81(100), 71(95), 95(83), 91(77), 55(52), 41(47), 67(45), 82(42), 43(37), 123(34)
Acetaldehyde	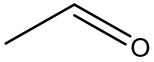	C_2H_4O	44(100), 43(53), 42(16)
Propanal	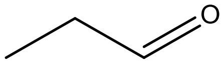	C_3H_6O	58(100), 57(50)
2-Propenal	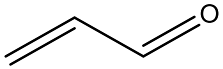	C_3H_4O	56(100), 55(74)
Butanal	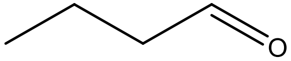	C_4H_8O	43(100), 44(98), 41(83), 72(53), 57(33), 82(18), 53(12), 42(11), 81(10)
2-methyl-Butanal	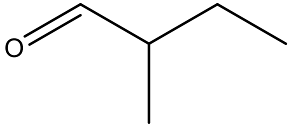	$C_5H_{10}O$	41(100), 57(89), 58(63), 43(10)
3-methyl-Butanal	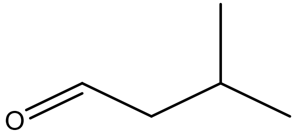	$C_5H_{10}O$	44(100), 41(99), 43(71), 58(61), 57(28), 71(21), 42(17), 45(12)

Hexanal	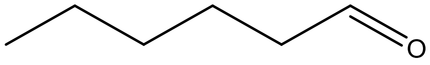	$C_6H_{12}O$	44(100), 56(91), 41(72), 57(61), 43(59), 72(21), 45(20), 55(17), 82(17), 67(15), 71(10), 58(10)
trans-2-Pentenal	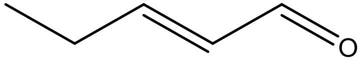	C_5H_8O	55(100), 83(71), 41(42), 84(27), 56(26), 53(19), 69(10)
Hex-3-enal	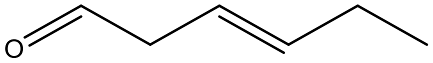	$C_6H_{10}O$	41(100), 69(31), 55(28), 70(15), 80(14), 83(11)
Pentanal	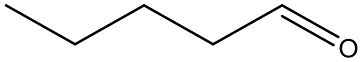	$C_5H_{10}O$	44(100), 70(76), 43(74), 41(55), 55(54), 57(47)
trans-Hex-2-enal	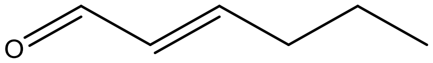	$C_6H_{10}O$	41(100), 55(96), 69(79), 83(68), 42(62), 57(46), 70(28), 43(27), 56(23), 80(16), 97(14), 53(14), 54(11), 40(10), 79(10)
Octanal	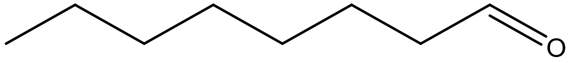	$C_8H_{16}O$	41(100), 57(97), 43(91), 44(75), 55(74), 56(68), 84(61), 82(47), 69(46), 42(43),
Nonanal	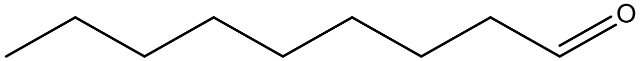	$C_9H_{18}O$	57(100), 41(83), 56(74), 55(59), 43(53), 70(45), 44(36), 95(35), 68(34),67(33), 81(32), 69(29), 82(27), 98(23),45(19), 42(17), 71(15), 96(14), 83(11)
trans,*trans*-Hexa-2,4-dienal	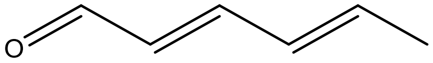	C_6H_8O	81(100), 53(34), 41(34), 96(28), 67(25, 82(20)
trans,*trans*-Hepta-2,4-dienal	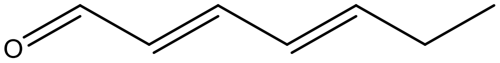	$C_7H_{10}O$	81(100), 53(19), 41(16), 79(13), 110(13), 67(11)

Heptadecanal	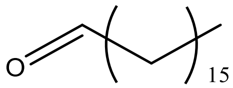	$C_{17}H_{34}O$	43(100), 57(85), 55(78), 82(78), 41(70), 67(54),81(51), 83(50), 96(46), 68(46), 71(44), 95(40), 69(36), 85(31), 70(30), 97(25), 56(23), 110(18), 84(18), 44(18)
1-Penten-3-ol	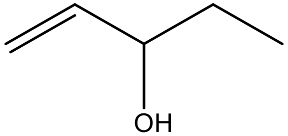	$C_5H_{10}O$	57(100), 41(6), 43(6)
1-pentanol	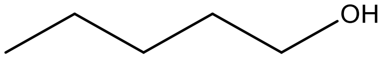	$C_5H_{12}O$	42(100), 55(89), 41(59), 70(36)
cis-2-Penten-1-ol	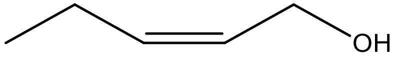	$C_5H_{10}O$	57(100), 44(22), 41(21), 43(18), 68(16), 67(15), 55(13), 53(10)
1-Hexanol	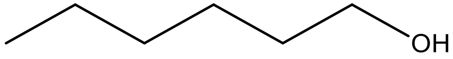	$C_6H_{14}O$	56(100), 43(59), 55(49), 41(43), 42(37), 69(35)
trans-3-Hexenol	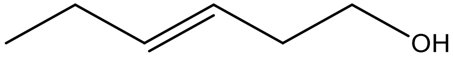	$C_6H_{12}O$	41(100), 67(91), 55(44), 82(42), 69(28), 42(23),53(16), 57(14), 54(12)
trans-2-Hexen-1-ol	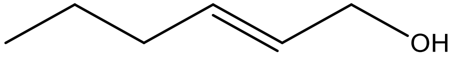	$C_6H_{12}O$	57(100), 41(57), 67(23), 82(21), 43(16), 44(14), 56(13), 55(12), 71(11)
1-Octen-3-ol	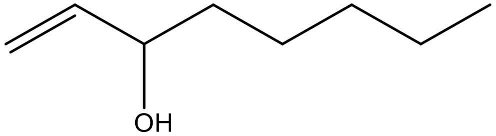	$C_8H_{16}O$	57(100), 43(22), 72(13), 41(12), 55(11)
1-Octanol	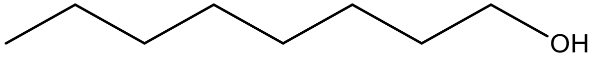	$C_8H_{18}O$	56(100), 55(82), 41(54), 42(53), 57(44), 70(41), 69(36), 84(35), 43(29), 83(20), 59(16), 85(13),

3,4-Dimethylcyclohexanol	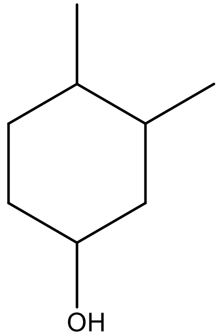	$C_8H_{16}O$	71(100), 43(71), 95(70), 57(33), 58(30), 41(24), 110(21), 85(18))
3,5,5-Trimethyl-hex-2-ene	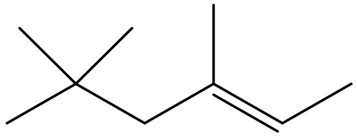	C_9H_{18}	100(57), 38(70), 55(32), 41(27), 69(17), 42(10), 109(10)
2-Ethylfuran	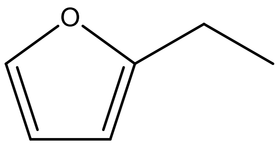	C_6H_8O	81(100), 96(41), 53(31),
2-pentyl-Furan	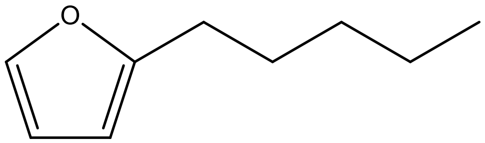	$C_9H_{14}O$	81(100), 82(24), 138(20), 53(13)
Furfural	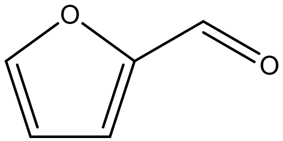	$C_5H_4O_2$	95(100), 96(99), 67(13)
2-Butanone	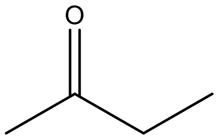	C_4H_8O	43(100), 72(31), 57(12)

3-methyl-2-Butanone	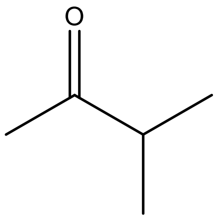	$C_5H_{10}O$	43(100), 86(21), 41(19), 44(12), 49(11)
2,3-Butanedione	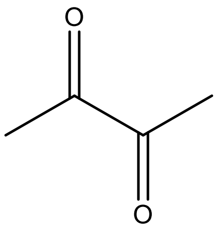	$C_4H_6O_2$	43(100), 86(20)
2,3-Pentanedione	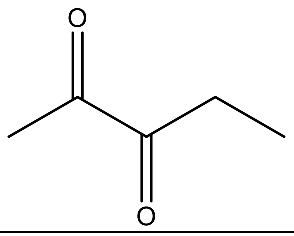	$C_5H_8O_2$	43(100), 57(58), 100(18)
3-Octanone	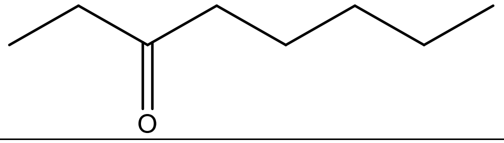	$C_8H_{16}O$	43(100), 57(71), 71(53), 99(47), 71(37), 41(24), 55(18), 68(11), 56(10)
Acetoin	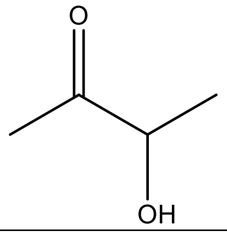	$C_4H_8O_2$	45(100), 43(60), 88(10)

D-Limonene	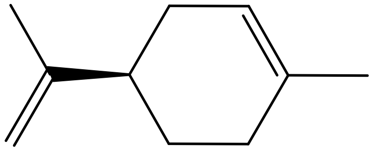	$C_{10}H_{16}$	68(100), 67(99), 93(95), 94(44),79(39), 92(32), 91(30), 121(27), 107(25), 53(23), 77(20), 41(17), 80(15), 136(12), 81(11)
β-Cymene	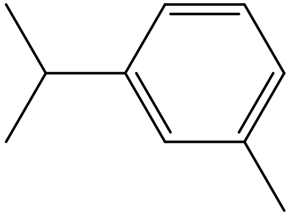	$C_{10}H_{14}$	119(100), 91(30), 134(28), 94(25), 117(21), 66(11), 115(11)
Acetol	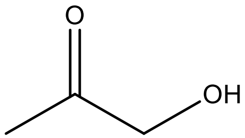	$C_3H_6O_2$	43(100), 74(9)
Benzaldehyde	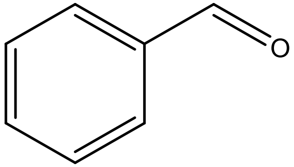	C_7H_6O	105(100), 106(97), 77(93), 51(44), 50(23), 78(13)
Linalool	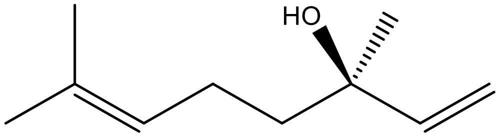	$C_{10}H_{18}O$	71(100), 93(73), 43(51), 41(47), 55(47), 69(37), 80(34), 121(19), 92(19), 91(15), 81(14), 67(13), 83(13), 79(12). 53(12), 41(10)
1,3,4-trimethyl-Cyclohexene-1-carboxaldehyde	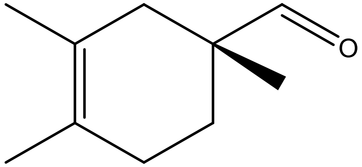	$C_{10}H_{16}O$	81(100), 109(81), 137(74), 123(74), 91(69), 152(64), 95(48), 41(47)
Benzeneacetaldehyde	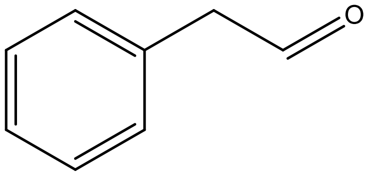	C_8H_8O	91(100), 92(30), 65(20), 120(15)

Methyl salicylate	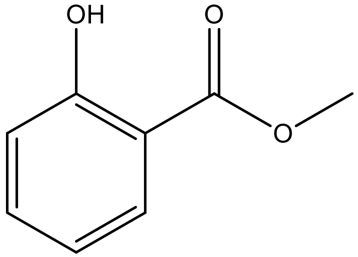	$C_8H_8O_3$	120(100), 92(76), 152(58), 121(32), 65(21), 93(21)
6,10-Dimethyl-5,9-undecadien-2-one	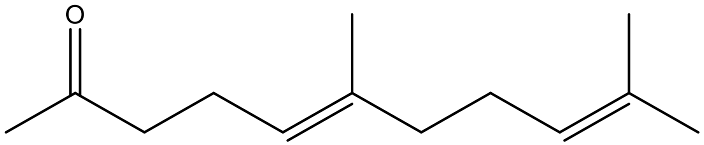	$C_{13}H_{22}O$	43(100), 69(41), 41(32), 107(18), 136(17)
β-Ionone	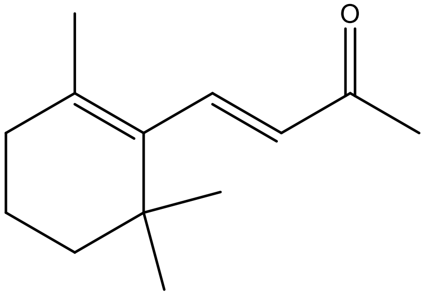	$C_{13}H_{20}O$	177(100), 43(39), 91(16), 44(14), 133(12), 107(11), 105(10)
4-Vinylguaiacol	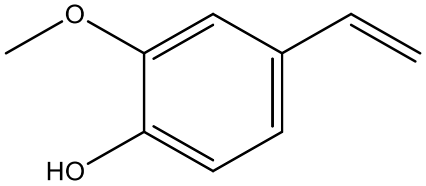	$C_9H_{10}O_2$	150(100), 135(97), 107(52), 77(45), 79(29), 151(15), 78(11)
Phytol	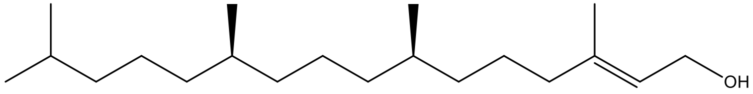	$C_{20}H_{40}O$	71(100), 45(36), 57(36), 43(30), 81(27), 55(27), 69(23), 41(21), 123(19), 68(17), 70(17)

Supplementary Figure 6. Leaf consumption (cm^2) in *Glycine max* leaves of ten cultivars in an *in vivo* bioassay. The data are based on ten replicas. Standard errors are displayed.

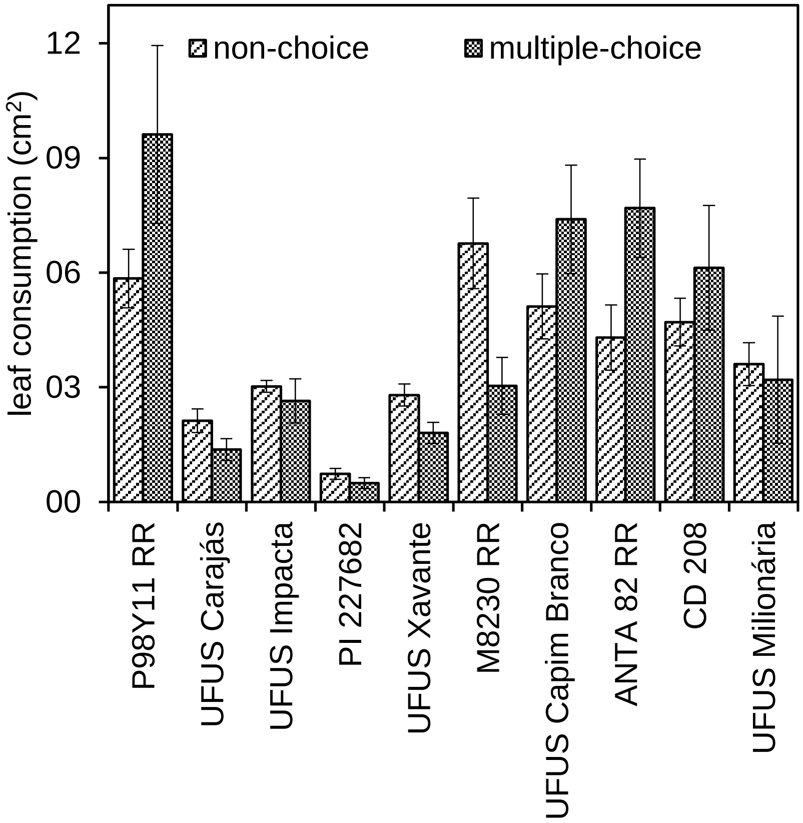

Supplement: Supplementary file 1 [file Data_Sheet_1.pdf]
